# Exendin-4 protects brain endothelial cell damage against hyperammonemic condition

**DOI:** 10.1016/j.bbrep.2026.102644

**Published:** 2026-05-22

**Authors:** Seo Yeon Ahn, Danbi Jo, Seo Yoon Choi, Juhyun Song

**Affiliations:** a Department of Anatomy, Chonnam National University Medical School, Hwasun, 58128, Republic of Korea; b Biomedical Science Graduate Program (BMSGP), Chonnam National University, Hwasun, 58128, Republic of Korea

**Keywords:** Hepatic encephalopathy (HE), Blood-brain barrier (BBB), Hyperammonemia, Exendin-4, Glucagon like peptide 1 (GLP-1)

## Abstract

Hepatic encephalopathy (HE) is a neuropsychiatric disorder associated with elevated ammonia levels, which impair blood-brain barrier (BBB) integrity and trigger chronic neuroinflammation. This study aims to evaluate the therapeutic potential of exendin-4, a glucagon-like peptide-1 receptor agonist, in mitigating BBB disruption and inflammation induced by hyperammonemia. We conducted experiments using a HE mouse model and *in vitro* cell culture systems. Using a bile duct ligation mouse model of HE, hepatic impairment was confirmed through significantly elevated serum aspartate aminotransferase and alanine aminotransaminase levels. Additionally, molecular cerebral changes were observed, including reduced tight junction protein expression and increased markers of oxidative stress and apoptosis. In an *in vitro* hyperammonemia model, we found that high ammonia levels significantly increased cell permeability, decreased tight junction protein expression (such as claudin-5) in bEnd.3 endothelial cells, and elevated pro-inflammatory cytokine secretion in C8-D1a astrocytes. Exendin-4 treatment effectively reversed these alterations, preserving BBB integrity and suppressing pro-inflammatory cytokine release. RNA sequencing further revealed transcriptional alterations in bEnd.3 endothelial cells after exendin-4 treatment. These findings highlight the potential of exendin-4 to mitigate hyperammonemia-induced BBB dysfunction, supporting its therapeutic value in treating neuropathologies associated with HE.

## Highlights

• Bile duct ligation surgery aggravates blood brain barrier disruption and cell apoptosis.
• Exendin-4 protects cell permeability in brain endothelial cells under hyperammonia condition.
• Exendin-4 protects tight junction protein loss in brain endothelial cells and astrocyte dysfuntion
• Exendin-4 affects genetic changes in brain endothelial cells under hyperammonia condition.

## Graphical abstract

**Figure:**
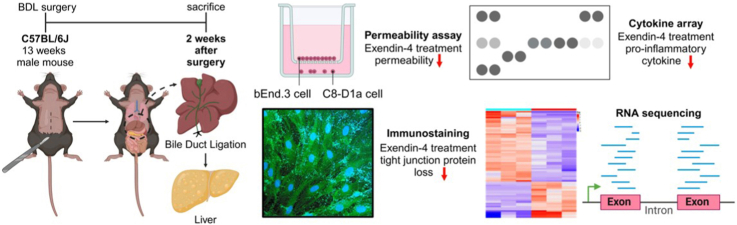

## 1 Introduction

Hepatic encephalopathy (HE) is a neuropsychiatric syndrome resulting from liver insufficiency and/or portosystemic shunts [1]. It manifests as a spectrum of neuropsychiatric disturbances, ranging from cognitive and motor dysfunction to coma and astrocytic edema. HE is characterized by elevated ammonia levels in the blood and cerebrospinal fluid (CSF), along with systemic inflammation and oxidative stress [[2], [3], [4], [5]]. Acute HE involves cerebral edema, increased intracranial pressure, and cerebral hemorrhage, with a high mortality rate (55–70%) [6]. Hyperammonemia compromises blood-brain barrier (BBB) integrity, reduces tight junction protein expression, and induces oxidative stress and neuroinflammation, contributing to neuropathological conditions, including memory loss and motor dysfunction [[7], [8], [9]]. The BBB is a physiological barrier composed of cerebrovascular endothelial cells, pericytes, and astrocytes. It serves as an interface between the central nervous system (CNS) and peripheral circulation, regulating ionic balance, facilitating nutrient transport, and restricting harmful molecules [10]. BBB disruption contributes to the progression of various neurological diseases [10]. Preventing BBB breakdown under hyperammonemic conditions is a key therapeutic target for mitigating neuropathologies in patients with HE.

Glucagon-like peptide-1 (GLP-1) receptor agonists, such as exendin-4, are known to enhance glucose metabolism, improve insulin sensitivity, regulate food intake, and reduce hepatic steatosis and liver cirrhosis [[11], [12], [13], [14], [15], [16]]. In the CNS, exendin-4 protects brain tissue in neurological conditions such as cerebral ischemia [17]. Our previous studies demonstrated that exendin-4 suppresses neuroinflammation [18] and improves mitochondrial function, neurite outgrowth, brain insulin resistance [19], and long-term potentiation [20]. Other studies suggest that GLP-1 and its receptor agonists help maintain BBB integrity and prevent its breakdown by modulating brain endothelial cell and astrocyte function [21,22].

Therefore, this study aims to investigate the effects of the GLP-1 receptor agonist exendin-4 on BBB component cells, including bEnd.3 cells and C8-D1a astrocytes, under ammonia-induced toxicity. We examined brain tissue changes in a bile duct ligation (BDL) mouse model of HE. The effect of exendin-4 on bEnd.3 cells and C8-D1a astrocytes against ammonia-induced toxicity were also assessed by measuring cell morphology, cytokine levels, mRNA expression, and protein levels. Furthermore, RNA sequencing was performed to identify genetic alterations in exendin-4-treated bEnd.3 cells under ammonia-induced toxicity. The findings of this study could highlight the potential protective effect of exendin-4 against BBB disruption in patients with HE.

## 2 Materials and methods

### 2.1 BDL surgery for hepatic encephalopathy model

Thirteen-week-old male C57BL/6 mice (n = 3 each group, Damul Science, Seoul, Republic of Korea) were used. Upon arrival, they were housed in the Laboratory Animal Research Center of Chonnam National University under controlled conditions: a 16 h light/8 h dark cycle, = 23 °C ambient temperature, 60 ± 10% humidity, and ad libitum access to water and food. After a 1 week acclimation, mice were randomly assigned to the BDL or SHAM group. The HE model was established using BDL, following previously described methods. Mice were anesthetized with an intraperitoneal injection of 2,2,2-tribromoethanol/2-methyl-2-butanol (Sigma-Aldrich, St. Louis, MO, USA) at 0.2 mg/g body weight. The surgical procedure was conducted under 1.5–2.0% isoflurane anesthesia in a mixture of air and oxygen. A midline abdominal incision was made to expose the bile duct, which was carefully isolated and doubly ligated with sterile 5-0 silk sutures. The bile duct was then transected between the ligatures to obstruct bile flow. In the SHAM group, mice underwent identical procedures without BDL or tran-section. The abdominal wall and skin were closed in two layers using absorbable and nonabsorbable sutures, respectively. Following surgery, each mouse was housed individually to facilitate recovery and prevent complications. Daily observations were conducted to assess pain, weight loss, or signs of distress, and supportive care was provided as needed for 2 weeks until euthanasia. Before euthanasia, mice were deeply anesthetized with 2-3% isoflurane delivered via inhalation. Anesthesia depth was confirmed through the absence of pedal withdrawal reflex. Mice were euthanized via cervical dislocation under deep anesthesia. All experimental procedures adhered to the "’96 Guidance for Animal Experiments” and were approved by the Animal Ethics Committee of Chonnam National University (CNU), protocol number No. CNU IACUC-H-2022-8, approved March 14, 2022.

### 2.2 Cell culture, Co-culture, and drug treatment

bEnd.3 cells and C8-D1a astrocytes were cultured and assigned to four experimental groups: Ctr, ammonia (10 mM; NH_3_/NH_4_^+^), exendin-4 (10 nM), and ammonia plus exendin-4. bEnd.3 cells were seeded on Transwell inserts (0.4 μm pore size) (Merck, ECM642; Burlington, MA, USA) to model the BBB microenvironment, while C8-D1a cells were seeded in standard culture plates. Cells were maintained in Dulbecco's Modified Eagle Medium (ATCC, Manassas, VA, USA) supplemented with 10% fe-tal bovine serum (Millipore, Billerica, MA, USA), 100 U/mL penicillin–streptomycin, and 1 mM sodium pyruvate (Thermo Fisher Scientific, Waltham, MA, USA) at 37 °C in a humidified 5% CO_2_ atmosphere.

Cells were treated with ammonium chloride (NH_4_Cl, Sigma) to mimic hyperammonemic conditions. Based on previous studies that used 3–20 mM NH_4_Cl to induce cellular stress in astrocytes, endothelial cells, or brain slice cultures [23,24], we selected 10 mM as a stringent concentration to model severe hyperammonemia. At pH 7.4 (pKa ≈9.25), this corresponds to approximately 0.14 mM free NH_3_, which has been shown to be sufficient to elicit cellular responses relevant to BBB dysfunction. For the coculture system, bEnd.3 cells were seeded in the upper chamber of Transwell inserts, while C8-D1a cells were seeded in the lower wells to model the BBB. When cells reached approximately 80% confluence, exendin-4 was added to the ammonia plus exendin-4 group 2 h before ammonia exposure. This 2-h pre-treatment window was strategically chosen based on previous *in vitro* studies to allow sufficient time for GLP-1 receptor binding and the activation of intracellular protective signaling pathways prior to the onset of severe hyperammonemic stress. The concentration of 10 nM exendin-4 was selected based on prior established protocols demonstrating optimal GLP-1 receptor activation and neuroprotection without inducing cytotoxicity in brain cells. Treatments were applied for 24 h. The coculture system was then used for a FITC-dextran permeability assay (70 kDa, 1 mg/mL final concentration) from the In Vitro Vascular Permeability Assay Kit (Merck, ECM644; Burlington, MA, USA) to assess the effects of hyperammonemia and exendin-4 on BBB integrity.

Following treatment, bEnd.3 cells were harvested for RNA and protein analyses to examine changes in tight junction proteins and genes related to BBB integrity. Similarly, C8-D1a cells underwent RNA and protein analyses, and their supernatants were collected for cytokine profiling using a multiplex cytokine array to evaluate inflammatory responses. All experiments were performed in triplicate to ensure reliability and re-producibility.

### 2.3 Cytokine array analysis

Supernatants from treated C8-D1a astrocytes were collected and stored at −80 °C for cytokine analysis. Cytokine expression was analyzed using a multiplex cytokine array kit (R&D Systems, ARY006, Minneapolis, MN, USA) following the instructions of the manufacturer. Additionally, 100 μL of each supernatant was incubated with pre-coated antibody arrays specific for pro- and anti-inflammatory cytokines. After unbound proteins were removed through multiple washing steps, fluorescently labeled secondary antibodies were added to detect captured cytokines. Fluorescence signals were measured at specific excitation/emission wavelengths of 485/535 nm using a plate reader. Raw fluorescence intensities were normalized to background using Chemi Reagent Mix and analyzed with Fusion Solo software (Vilber, Collégien, France).

### 2.4 RNA sequencing analysis

RNA samples were prepared for RNA-seq analysis from bEnd.3 cells treated with ammonia or ammonia plus exendin-4. Total RNA was extracted using RNAiso Plus (Takara, Shiga, Japan) and assessed for integrity with the 2100 Bioanalyzer System (Agilent Technologies, Santa Clara, CA, USA). RNA-seq libraries were constructed using the TruSeq Stranded Total RNA kit (Illumina, San Diego, CA, USA) and NovaSeq 6000 system (Illumina, San Diego, CA, USA. Reads were pseudo-aligned and quantified with Kallisto to obtain transcript-level counts and TPMs [25]. Gene-level abundances were calculated according to the GENCODE mouse annotation. Genes with average TPM <1 across samples or with TPM = 0 in any sample were excluded, leaving 10,681 genes for testing. Between-sample normalization was performed on gene-level counts using TMM. For each gene, differential expression between the two groups (n = 3 per group) was assessed by a two-sample *t*-test. To control for multiple testing across the 10,681 genes, p-values were adjusted using the Benjamini–Hochberg procedure (FDR); transcriptome-wide significance was defined as FDR <0.05. In parallel, for hypothesis-generating prioritization, we examined genes with unadjusted p < 0.05 and ranked them by fold change to nominate candidates for downstream validation.

### 2.5 Genes with altered expression

From the exploratory screen (unadjusted p < 0.05), 425 genes showed altered expression in ammonia plus exendin-4–treated cells relative to ammonia alone (183 increased and 242 decreased). Volcano plots display log_2_ (fold change) versus –log_10_(unadjusted p) and are explicitly labeled as exploratory. From these, the top 20 genes with increased expression and the top 20 with decreased expression were selected based on fold change. For enrichment analyses, we used the filtered transcriptome (10,681 genes) as the background universe. Gene Ontology (GO) and Kyoto Encyclopedia of Genes and Genomes (KEGG) over-representation analyses were performed on the top 400 exploratory candidates (ranked by unadjusted p among those with p < 0.05) using the Molecular Signatures Database [26]. The top 10 enriched GO terms and KEGG pathway terms were selected based on the FDR *q*-value. For the 10 protein-coding genes with the highest increase and decrease in fold change among those with a p-value <0.05, gene–gene association networks were explored in Cytoscape (v3.9.1) with the GeneMANIA plugin. Cytoscape is an open-source platform developed by the National Human Genome Research Institute, Ontario, Canada.

### 2.6 RNA extraction and quantitative reverse transcription PCR (qRT-PCR)

Total RNA was extracted from brain tissues and cultured cells using TRIzol Reagent (Ambion, Austin, TX, USA) following the guidelines of the manufacturer. RNA purity and concentration were determined by measuring the 260/280 nm absorbance ratio with a NanoDrop spectrophotometer (Implen, Westlake Village, CA, USA). cDNA was synthesized from 1 μg of RNA using a reverse transcription kit (Invitrogen, Carlsbad, CA, USA) according to the protocol of the manufacturer. Quantitative PCR was performed on a real-time PCR system with SYBR Green Master Mix (Enzynomics, RT501 M, Seoul, Republic of Korea). Gene-specific primers were designed using Primer-BLAST. The amplification protocol included an initial denaturation at 95 °C for 10 min, followed by 40 cycles of 95 °C for 15 s and 60 °C for 60 s. Relative gene expression was calculated using the 2^−^ΔΔCt method and normalized to GAPDH. Supplementary Table 1 lists all RT-PCR primers.

### 2.7 Western blot analysis

Proteins were extracted from brain tissues and cultured cells using RIPA lysis buffer (Translab, Daejeon, Republic of Korea) supplemented with 1× protease and 1× phosphatase inhibitor cocktails. Protein concentration was measured using a BCA assay kit (Thermo Fisher Scientific, Waltham, MA, USA) following the instructions of the manufacturer. Equal amounts of protein (30 μg) were separated on 10% sodium dodecyl sulfate-polyacrylamide gels and transferred onto PVDF membranes using a wet transfer system. Membranes were blocked with 5% skim milk in Tris-buffered saline containing 0.1% Tween-20 (TBS-T) for 1 h at room temperature. After blocking, Membranes were incubation with primary antibodies (1:1000 dilution in TBS-T) at 4 °C overnight. After incubation, membranes were incubated with HRP-conjugated secondary antibodies (mouse anti-rabbit IgG or goat anti-mouse IgG; Santa Cruz Biotechnology, Dallas, TX, USA) at a 1:5000 dilution in 1× TBS-T for 2 h at room temperature. Protein bands were visualized using enhanced chemiluminescence (ECL) reagents (Thermo Fisher Scientific, Waltham, MA, USA) and imaged with the Fusion Solo imaging system (Vilber, Collégien, France). The following primary antibodies were used: Claudin-5 (Abcam, Cambridge, UK; ab15106), Occludin (Santa Cruz Biotechnology, Dallas, TX, USA; sc133256), postsynaptic density-95 (PSD-95) (Cell Signaling Technology, Danvers, MA, USA; 3409s), synaptophysin (SYP) (Cell Signaling Technology, Danvers, MA, USA; 36406s), glucagon like peptide 1 receptor (GLP-1R)(Santa Cruz Biotechnology, Dallas, TX, USA; sc390774), doublecortin (DCX) (Abcam, Cambridge, UK ab18723), adenosine a2a receptor (AR2R) (Abcam, Cambridge, UK; ab3461), (Santa Cruz Biotechnology, Dallas, TX, USA; sc393859), glial fibrillary acidic protein (GFAP) (Santa Cruz Biotechnology, Dallas, TX, USA; sc33673), hypoxia inducible factor 1 alpha (HIF-1α) (Cell Signaling Technology, Danvers, MA, USA; 14179S), and GAPDH (Santa Cruz Biotechnology, Dallas, TX, USA; sc32233). The specificity of the primary antibodies was further validated using secondary antibody-only controls, which showed no detectable bands. Membranes were then washed with TBS-T and incubated with HRP-conjugated secondary antibodies for 1 h at room temperature. Chemiluminescent signals were detected using an ECL substrate and visualized with a digital imaging system. Protein band intensities were quantified with ImageJ software. Target protein levels were normalized to GAPDH, phosphorylated proteins to their corresponding unphosphorylated forms, and cleaved proteins to their full-length forms to ensure consistent analysis.

### 2.8 Cell permeability assay

BBB permeability was evaluated using the In Vitro Vascular Permeability Assay Kit (Merck, ECM644, Burlington, MA, USA). In the coculture model, bEnd.3 cells were seeded onto collagen-coated Transwell inserts (1 μm pore size), while C8-D1a astrocytes were cultured in the corresponding wells of a 24-well plate to replicate BBB conditions. After treatments, FITC-dextran (70 kDa; final concentration, 1 mg/mL) was added to the upper chamber of the Transwell system and incubated at 37 °C for 2 h. A 100-μL aliquot was then collected from the lower chamber for fluorescence measurement. Fluorescence intensity was measured using a microplate reader (excitation/emission, 485/535 nm). The permeability coefficient was determined by comparing the fluorescence intensity of the lower chamber with that of the upper chamber. Results were normalized to the control group and expressed as a percentage to evaluate treatment effects on BBB integrity.

### 2.9 Monolayer staining

bEnd.3 cells cultured on collagen-coated Transwell inserts (1 μm pore size) from the In Vitro Vascular Permeability Assay Kit (ECM644, Merck, Burlington, MA, USA) were fixed with 4% paraformaldehyde for 10 min at room temperature. After fixation, monolayers were washed thrice with phosphate-buffered saline (PBS) to remove residual fixative. Cells were permeabilized with 0.1% Triton X-100 in PBS for 5 min and rinsed with PBS. To reduce nonspecific binding, blocking was performed with 5% bovine serum albumin (BSA) in PBS for 1 h at room temperature. For nonfluorescent staining, the monolayers were incubated with 0.5% (w/v) crystal violet in 20% methanol for 10 min. Excess stain was removed with distilled water, and the monolayers were air-dried before light-microscopy imaging to assess cell morphology and monolayer integrity. This process facilitated the assessment of bEnd.3 monolayer conditions under different experimental treatments.

### 2.10 Immunocytochemistry for protein localization

bEnd.3 cells cultured on sterilized glass coverslips were fixed with 4% paraformaldehyde for 15 min at room temperature and then washed thrice with PBS. Cells were permeabilized with 0.1% Triton X-100 in PBS for 10 min. Nonspecific binding was blocked by incubating the cells in 5% BSA in PBS for 1 h. Primary antibodies against claudin-5 (1:500) (Abcam, Cambridge, UK; ab15106) and occludin (1:500)(Santa Cruz Biotechnology, Dallas, TX, USA; sc133256) were diluted in 1% BSA and incubated overnight at 4 °C. To ensure the specificity of the immunostaining, negative controls were performed for each experiment by omitting the primary antibodies and incubating the samples with secondary antibodies only. These controls exhibited no significant fluorescence signal. After washing with PBS, the coverslips were incubated with fluorescently labeled secondary antibodies (Alexa Fluor 488 donkey anti-rabbit IgG [H + L], Thermo Fisher Scientific, Waltham, MA, USA, A21202; Alexa Fluor 594 donkey anti-mouse IgG [H + L], Thermo Fisher Scientific, Waltham, MA, USA, A21203) for 1 h in the dark. Nuclei were counterstained with 4′,6-diamidino-2-phenylindole (DAPI; 1 μg/mL) for 5 min. Coverslips were then mounted onto glass slides using an antifade mounting medium and imaged with a K1-Fluo confocal microscope (Nanoscope Systems, Daejeon, Republic of Korea) at 40× magnification. Fluorescence intensity was quantified using ImageJ software (National Institutes of Health, Bethesda, MD, USA). For fluorescence quantification, at least five random fields were captured per sample. Fluorescence intensity was measured using ImageJ by calculating the mean fluorescence intensity after subtracting the background signal, which was determined from regions without cells. Data were normalized to the control group and expressed as a fold change. The fluorescence intensity (or protein level) was expressed as a relative fold change compared to the control group, with the control value defined as 1.

### 2.11 Mitochondrial membrane potential and ROS detection

To evaluate mitochondrial function and oxidative stress in bEnd.3 cells, JC-1 and DCF-DA assays were performed. Cells were divided into four groups: Ctr, ammonia (10 nM NH_3_/NH_4_^+^), exendin-4 (10 nM), and ammonia plus exendin-4. Treatments were conducted for 24 h as described above. Mitochondrial membrane potential (MMP) was assessed using the JC-1 Mitochondrial Membrane Potential Assay Kit (Abcam, ab113850, Cambridge, UK) according to the instructions of the manufacturer. Briefly, treated bEnd.3 cells were incubated with JC-1 dye (5 μg/mL) for 30 min at 37 °C in the dark. Following incubation, cells were washed twice with assay buffer and mounted in DAPI-containing mounting medium. JC-1 aggregates (red, high MMP) and JC-1 monomers (green, low MMP) were visualized under a confocal microscope (K1-Fluo, Nanoscope Systems, Daejeon, Republic of Korea) using identical settings. The fluorescence intensity ratio of green to red (monomer/aggregate) was quantified using ImageJ software to assess mitochondrial depolarization. Intracellular ROS levels were assessed using the DCF-DA Cellular ROS Detection Assay Kit (Abcam, ab113851). bEnd.3 cells were incubated with 25 μM DCF-DA for 45 min at 37 °C in the dark, washed with PBS, and immediately imaged using the same confocal microscope under green fluorescence excitation/emission (Ex/Em: 485/535 nm). Fluorescence intensity was quantified using ImageJ software, and increased fluorescence indicated elevated intracellular ROS production. All experiments were independently repeated at least three times. For fluorescence quantification, at least five random fields were captured per sample. Fluorescence intensity was measured using ImageJ by calculating the mean fluorescence intensity after subtracting the background signal, which was determined from regions without cells. Data were normalized to the control group and expressed as a fold change. Quantitative data were expressed as mean ± standard error of the mean (SEM). The fluorescence intensity (or protein level) was expressed as a relative fold change compared to the control group, with the control value defined as 1. Statistical significance was determined using one-way analysis of variance (ANOVA) followed by Tukey's post hoc test, with *p* < 0.05 considered significant.

### 2.12 Statistical analysis

Data are presented as the mean ± SEM from at least three independent experiments. Comparisons between two groups were performed using an unpaired two-tailed *t*-test with Welch's correction. For comparisons among multiple groups, a one-way ANOVA followed by Tukey's post hoc test was used. Statistical significance was defined as ∗*p* < 0.05, ∗∗*p* < 0.01, ∗∗∗*p* < 0.001, and ∗∗∗∗*p* < 0.0001.

## 3 Results

### 3.1 BDL operation induces regional BBB disruption and molecular alterations in the cortex and hippocampus

**Fig. 1:**
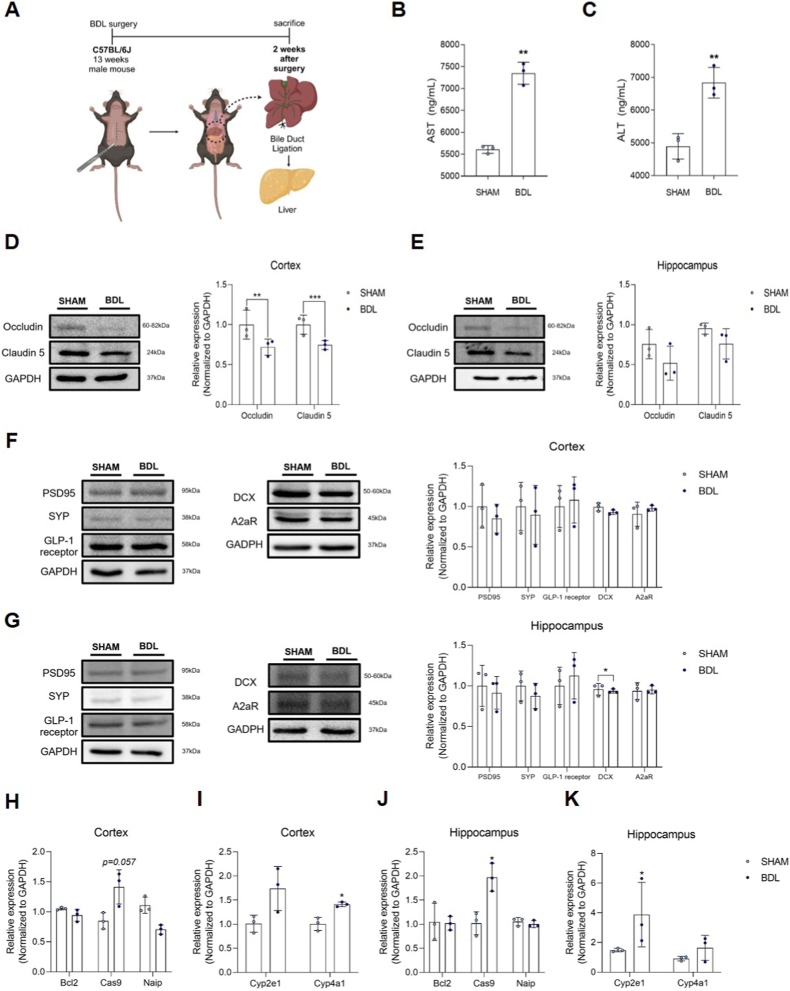
**Protein and mRNA levels in the cortex and hippocampus of the BDL mouse.** (A) Schematic diagram of the BDL operation. (B) Plasma AST levels in the BDL mouse. (C) Plasma ALT levels in the BDL mouse. (D, E, F, G) Protein levels (Western blot) (D) Tight junction protein expression in the cortex. (E) Tight junction protein expression in the hippocampus. In the hippocampus, occludin and claudin 5 showed a reduction trend in BDL mice compared with sham, but the differences were not statistically significant. (F) Protein levels in the cortex. (G) Protein expression in the hippocampus. Western blot analysis of PSD, SYP, GLP-1, and Bcl2 in cortex, hippocampus, and striatum. Although slight differences in band intensity were observed, these proteins did not show statistically significant changes between sham and BDL groups. (H, I, J, K) mRNA levels (RT-PCR) (H) mRNA levels of apoptosis-related genes in the cortex. (I) mRNA levels of ROS-related genes in the cortex. (J) mRNA levels of apoptosis-related genes in the hippocampus. (K) mRNA levels of ROS-related genes in the hippocampus. BDL, bile duct ligation; AST, aspartate aminotransferase; ALT, alanine aminotransferase; GAPDH, glyceraldehyde-3-phosphate dehydrogenase; PSD95, post synaptic density protein 95; SYP, synaptophysin; DCX, doublecortin; A2aR, adenosine A2a receptor; Bcl2, B-cell lymphoma 2; Cas9, caspase-9; Naip, NLR family apoptosis inhibitory protein; Cyp2e1, cytochrome P450 2E1; Cyp4a1, cytochrome P450 4A1; ROS, reactive oxygen species; ∗ *p*-value <0.05, ∗∗ *p*-value <0.01, ∗∗∗ *p*-value <0.001.

To assess changes in brain tissues and liver function in the HE model, we utilized the BDL surgical model, which is a well-established paradigm for chronic HE. Two weeks after BDL surgery on 13-week-old male C57BL/6J mice, we observed characteristic signs of cholestasis, hepatocyte injury, and fibrosis (Fig. 1A). Serum analysis confirmed extensive liver damage, as evidenced by significantly elevated levels of aspartate aminotransferase (AST) (Fig. 1B) and alanine aminotransferase (ALT) (Fig. 1C) compared to SHAM controls. Regarding the brain pathology, we observed regional differences in the response to BDL. In the cortex, protein levels of tight junction markers (Occludin and Claudin-5) and synaptic markers (PSD95 and SYP) were significantly reduced (Fig. 1D and F). In contrast, while the hippocampus showed a noticeable downward trend in the expression of Occludin, Claudin-5, PSD95, and SYP, these differences did not reach statistical significance after normalization to GAPDH (Fig. 1E and G). At the transcriptional level (mRNA), both the cortex and hippocampus exhibited significant increases in stress markers. However, at the translational level (protein), significant reductions were confined to the cortex, while the hippocampus showed only a downward trend. This regional disparity suggests that the cortical neurovascular unit may exhibit higher sensitivity or a more rapid pathological response to hyperammonemia compared to the hippocampal region at the 2-week time point. Despite the lack of significance in hippocampal protein levels, mRNA analysis revealed underlying molecular stress in both regions. In the BDL mouse brain tissue, mRNA levels of B-cell lymphoma 2 (Bcl2) were lower, while caspase-9 (Cas9) and cytochrome P450 family 2 subfamily E member 1 (Cyp2e1) were elevated in the cortex (Fig. 1H and I) and hippocampus (Fig. 1J and K), indicating enhanced apoptosis and oxidative stress.

Collectively, these findings suggest that BDL surgery induces significant liver damage and initiates a cascade of oxidative stress and apoptosis, alongside molecular alterations associated with neuroinflammation, leading to regional BBB disruption and synaptic dysfunction (Fig. 1).

### 3.2 Exendin-4 prevented cell permeability in bEnd.3 cells and the production of pro-inflammatory cytokines in C8-D1a cells under ammonia-induced toxicity

**Fig. 2:**
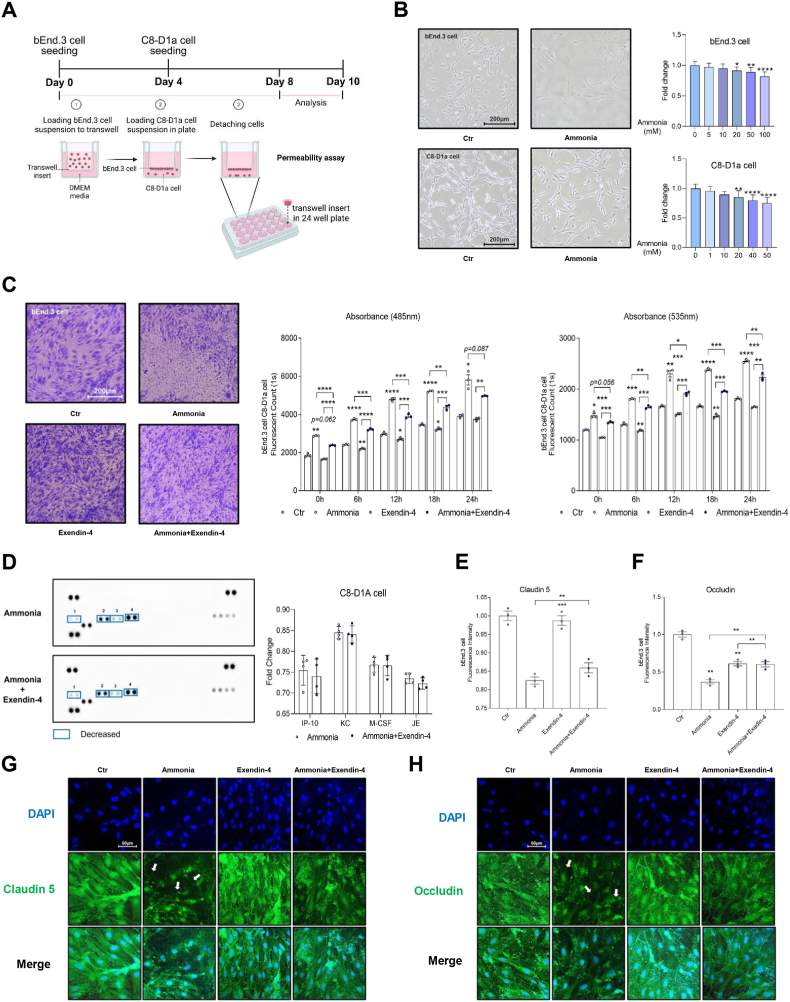
Effects of exendin-4 on bEnd.3 and C8-D1a under ammonia-induced toxicity. (A) Illustration of the *in vitro* study design. (B) Cell viability assay in bEnd.3 and C8-D1a cells after ammonia exposure. Ammonia sensitivity of bEnd.3 endothelial cells and C8-D1a astrocytes. Data are expressed as mean ± SEM. p < 0.05 vs control. Although slight differences in response patterns were observed between the two cell types, two-way ANOVA indicated that these differences were not statistically significant. (C) Permeability assay of exendin-4-treated bEnd.3 cells under ammonia-induced toxicity. (D) Cytokine array of exendin-4-treated C8-D1a cells under ammonia-induced toxicity. Cytokine levels after ammonia and exendin-4 treatment. Data are expressed as mean ± SEM. Although slight changes were observed, the differences were not statistically significant. (E) Fluorescence intensity of claudin-5 in all groups. (F) Fluorescence intensity of occludin in all groups. (G) Immunocytochemistry of claudin-5 in all groups. (H) Immunocytochemistry of occludin in all groups. Representative images are shown. Quantitative data in (E) and (F) were derived from the analysis of multiple random fields (n ≥ 5 per group) to ensure statistical reliability. Ctr, control group; IP-10, interferon gamma-induced protein 10 (CXCL10, C-X-C motif chemokine ligand 10); KC, keratinocyte-derived chemokine (CXCL1, C-X-C motif chemokine ligand 1); M-CSF, macrophage colony-stimulation factor (CSF1, Colony-stimulating factor 1); JE, monocyte chemoattractant protein-1, MCP-1 (CCL2, C–C motif chemokine ligand 2); DAPI, 4′6-diamidino-2-phenylinodole, blue color; FITC, green color; White arrow: tight junction protein loss, ∗ *p*-value <0.05, ∗∗ *p*-value <0.01, ∗∗∗ *p*-value <0.001, ∗∗∗∗*p*-value <0.0001.

To determine the effects of exendin-4 on the BBB, an *in vitro* BBB model was established using Transwell inserts, with bEnd.3 cells and C8-D1a astrocytes seeded on opposite sides (Fig. 2A). Ammonia treatment increased cell permeability and induced cellular damage in the *in vitro* BBB coculture system (Fig. 2A). Cell viability decreased in both cell types following ammonia exposure, with significant reductions observed at concentrations above 10 mM (Fig. 2B). Based on these results, 10 mM ammonia was selected for subsequent experiments. In Fig. 2B, bEnd.3 endothelial cells and C8-D1a astrocytes exhibited slight differences in their responses to ammonia exposure. However, two-way ANOVA analysis demonstrated that these differences were not statistically significant, indicating that the ammonia sensitivity of the two cell types was overall comparable. Compared to bEnd.3 cells, C8-D1a cells were more sensitive to ammonia-induced cytotoxicity (Fig. 2B). Permeability assays using FITC-dextran revealed increased permeability in ammonia-treated bEnd.3 cells, indicating junctional disruption (Fig. 2C). To further evaluate BBB integrity, permeability assays were conducted in a coculture system. bEnd.3 cells were seeded into Transwell inserts, while C8-D1a astrocytes were cultured in the corresponding wells to better stimulate the BBB microenvironment. The experiment included four groups: control (Ctr), ammonia, exendin-4, and ammonia plus exendin-4, enabling assessment of hyperammonemia and GLP-1 treatment effects on BBB permeability (Fig. 2C). Fluorescein isothiocyanate-dextran (FITC-dextran) was added to the upper chamber to measure BBB permeability, and its passage across the cell monolayer into the receiver tray was quantified through absorbance and fluorescence. Absorbance was measured at 485 nm to determine FITC-dextran concentration. The absorbance at this wavelength corresponds to the amount of light absorbed by the FITC-dextran molecules, which is directly proportional to their concentration. Higher absorbance indicates greater FITC-dextran accumulation and, consequently, increased permeability. In the ammonia-treated group, BBB disruption increased FITC-dextran permeability, reflected by elevated absorbance values over time. In contrast, the ammonia plus exendin-4 group exhibited lower absorbance, indicating that exendin-4 partially reduced ammonia-induced BBB damage by limiting FITC-dextran passage. Fluorescence was measured at 535 nm to assess FITC-dextran distribution across the BBB. Fluorescence corresponds to light emission after excitation and provides a quantitative measure of barrier integrity. Higher fluorescence intensity reflects increased FITC-dextran mobility, indicating BBB compromise. The ammonia-treated group exhibited the highest fluorescence values, confirming significant BBB disruption and enhanced FITC-dextran permeability. Conversely, the ammonia plus exendin-4 group showed lower fluorescence intensity than that of the ammonia-alone group, demonstrating the protective effect of exendin-4 in maintaining BBB integrity (Fig. 2C).

The combined assessment of absorbance at 485 nm and fluorescence at 535 nm provided a comprehensive measure of BBB permeability. For example, decreased absorbance alongside increased fluorescence indicates low FITC-dextran concentration but high mobility, suggesting partial barrier compromise. Conversely, simultaneous reductions in absorbance and fluorescence reflect strong BBB integrity. These findings underscore the utility of dual-wavelength measurements for accurately evaluating BBB function and the modulatory effects of exendin-4 on hyperammonemia-induced barrier disruption (Fig. 2C).

Cytokine profiling of C8-D1a astrocyte supernatants revealed that exendin-4 treatment under ammonia exposure reduced pro-inflammatory cytokines, including interferon gamma-induced protein 10 (IP-10), keratinocyte chemoattractant (KC), macrophage colony-stimulating factor (M-CSF), and monocyte chemotactic protein-1 (MCP-1, also known as JE) compared to those of the controls (Fig. 2D). Exendin-4 treatment showed a tendency to alter cytokine levels after ammonia exposure; however, these changes did not reach statistical significance (Fig. 2D). Immunocytochemistry revealed that tight junction proteins, such as claudin-5 (Fig. 2E and G) and occludin (Fig. 2F and H), were reduced in ammonia-treated bEnd.3 cells, but this loss was partially mitigated by exendin-4. Exendin-4 preserved claudin 5 (Fig. 2G and E) and occludin expression (Fig. 2F and H) in ammonia-treated bEnd.3 cells. Collectively, these findings demonstrate that exendin-4 significantly attenuates hyperammonemia-induced tight junction protein disruption, reduces endothelial cell permeability, and decreases astrocytic pro-inflammatory cytokine production in our *in vitro* models (Fig. 2).

### 3.3 Exendin-4 contributes to the expression of tight junction proteins on brain endothelial cells and the astrocyte activation

**Fig. 3:**
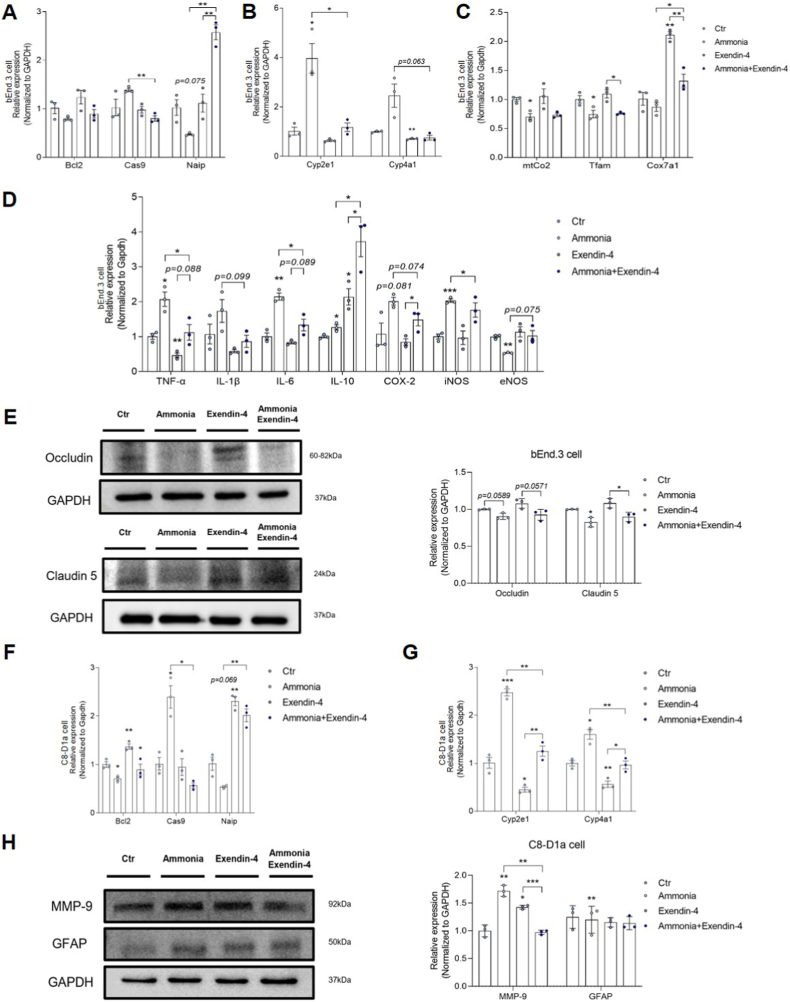
Protein and mRNA levels in exendin-4-treated bEnd.3 and C8-D1a cells under ammonia-induced toxicity. (A) mRNA levels of apoptosis-related genes in bEnd.3 cells. Bcl 2 expression after ammonia treatment. Data are expressed as mean ± SEM. Although a slight decrease in band intensity was observed, the change was not statistically significant. (B) mRNA levels of ROS-related genes in bEnd.3 cells. (C) mRNA levels of mitochondrial dysfunction-related genes in bEnd.3 cells. (D) mRNA levels of inflammatory cytokines in bEnd.3 cells. (E) Protein levels in bEnd.3 cells. (F) mRNA levels of apoptosis-related genes in C8-D1a cells. (G) mRNA levels of ROS-related genes in C8-D1a cells. (H) Protein levels in C8-D1a cells. Bcl2, B-cell lymphoma 2; Cas9, caspase-9; Naip, NLR family apoptosis inhibitory protein; Cyp2e1, Cytochrome P450 2E1; Cyp4a1, Cytochrome P450 4A1; mtCo2, mitochondrial cytochrome *c* oxidase subunit II; Tfam, mitochondrial transcription factor A; Cox7a1, cytochrome *c* oxidase subunit 7A1; TNF-α, Tumor Necrosis Factor-alpha; IL-1β, Interleukin-1 beta; IL-6, Interleukin-6; IL-10, Interleukin-10; COX-2, cyclooxygenase-2; iNOS, inducible nitric oxide synthase; eNOS, endothelial nitric oxide synthase; MMP-9, matrix metalloproteinase-9; GAPDH, glyceraldehyde-3-phosphate dehydrogenase; GFAP, glial fibrillary acidic protein, ROS, reactive oxygen species, ∗ *p*-value <0.05, ∗∗ *p*-value <0.01, ∗∗∗ *p*-value <0.001.

To evaluate the effects of exendin-4 on bEnd.3 cells and astrocytes, the mRNA levels of antioxidant markers and ROS-related genes were measured using reverse transcription PCR (RT-PCR) after treating cerebrovascular endothelial cells with exendin-4 under ammonia exposure (Fig. 3A–C). Ammonia exposure showed a slight tendency to reduce Bcl-2 expression; however, this difference was not statistically significant. Ammonia exposure reduced Bcl2 expression, accelerating cell death through mitochondrial dysfunction. However, exendin-4 pretreatment partially prevented this effect. Increased caspase-9 and Naip expression in cerebrovascular endothelial cells following ammonia exposure indicated heightened DNA damage and impaired antiapoptotic defenses. Exendin-4 significantly attenuated these changes, emphasizing its antiapoptotic activity (Fig. 3A). Hyperammonemia also elevated oxidative stress markers Cyp2e1 and Cyp4a1 (cytochrome P450 family 4 subfamily A member 1), whereas exendin-4 treatment reduced their expression (Fig. 3B). Ammonia exposure disrupted mitochondrial function, reducing the expression of mitochondrial cytochrome *c* oxidase subunit II, mitochondrial transcription factor A (Tfam), and cytochrome *c* oxidase subunit 7A1 (Cox7a1). However, exendin-4 mitigated these effects (Fig. 3C). Ammonia exposure also increased the expression levels of pro-inflammatory cytokines, including tumor necrosis factor-alpha, interleukin-1 beta, and interleukin-6 (IL-6), in brain vascular endothelial cells, while exendin-4 pretreatment significantly reduced their expression (Fig. 3D). Conversely, the anti-inflammatory cytokine interleukin-10 (IL-10) was significantly elevated in ammonia-exposed brain vascular endothelial cells following exendin-4 pretreatment, suggesting that exendin-4 promotes anti-inflammatory cytokine secretion (Fig. 3D). Additionally, ammonia-induced upregulation of cyclooxygenase-2 and inducible nitric oxide synthase, along with the downregulation of endothelial nitric oxide synthase, was significantly mitigated by exendin-4 pretreatment, indicating its role in restoring endothelial function (Fig. 3D). Ammonia exposure reduced the expression levels of tight junction proteins occludin and claudin-5, indicating BBB disruption. Exendin-4 treatment partially restored their expression in bEnd.3 cells relative to the ammonia-treated group (Fig. 3E). In C8-D1a cells, ammonia exposure decreased the mRNA expression of survival-related genes, including Bcl2, Cas9, and Naip (Fig. 3F), while increasing the expression of ROS-generating genes Cyp2e1 and Cyp4a1 (Fig. 3G). Exendin-4 pretreatment partially restored Bcl2 and Naip expression while decreasing Cas9, Cyp2e1, and Cyp4a1 levels, demonstrating its protective effects against ammonia-induced oxidative stress and cell death (Fig. 3F and G). Western blot analysis of matrix metalloproteinase-9 (MMP-9) and glial fibrillary acidic protein (GFAP) in C8-D1a cells revealed increased GFAP and MMP-9 protein levels, indicating astrocyte activation in response to ammonia-induced toxicity (Fig. 3H). Exendin-4 pretreatment reduced the expression of MMP-9, a marker of cell permeability, in ammonia-treated C8-D1a cells compared to those of the control group (Fig. 3H). Collectively, these biochemical assays show that exendin-4 pretreatment partially restores the expression of tight junction proteins and survival-related genes, while significantly mitigating ROS generation, pro-inflammatory cytokine expression, and MMP-9 elevation in ammonia-exposed endothelial cells and astrocytes.

### 3.4 Exendin-4 induces exploratory transcriptomic changes and modulates mitochondrial function and ROS generation in bEnd.3 cells under ammonia toxicity

**Fig. 4:**
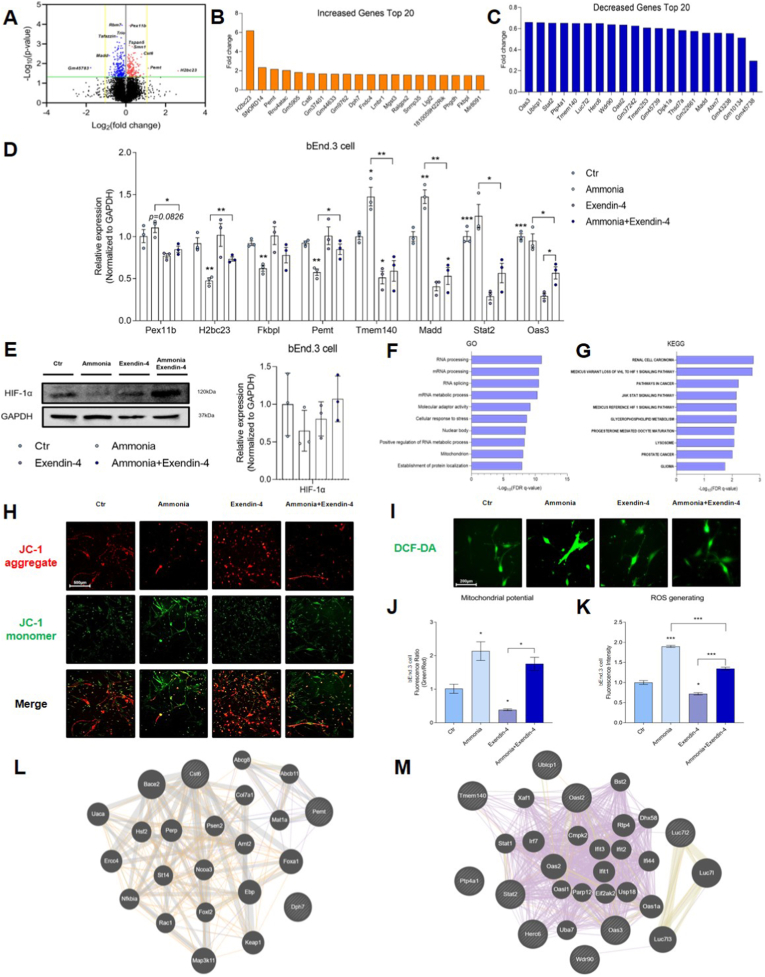
RNA-sequencing analysis, mitochondrial potential and ROS generation of exendin-4-treated bEnd.3 cells under ammonia toxicity. (A) Volcano plot of gene expression differences between the ammonia and ammonia + exendin-4 groups. Genes with p < 0.05 and ≥ 50% expression changes are highlighted. Yellow colored genes indicate upregulated genes (log_2_(HFD/NFD) > 1), whereas green colored genes indicate downregulated genes (-log_10_(p-value) >1.3 (B) Top 20 up-regulated candidates ranked by fold change among genes with unadjusted p < 0.05. (C) Top 20 down-regulated candidates ranked likewise. (D) Quantitative real time-PCR to measure mRNA level of several genes such as Pex11b, H2bc23, Fkbpl, Pemt, Tmem140, Madd, Stat2, and Oas3 genes. (E) Protein levels of HIF-1α in bEnd.3 cells under ammonia toxicity. (F) Gene Ontology (GO) analysis performed on the top 400 exploratory candidates using the filtered transcriptome (10,681 genes) as background. (G) Kyoto Encyclopedia of Genes and Genomes (KEGG) pathway analysis, same statistics and ranking as in (F). (H) Representative JC-1 fluorescence images of bEnd.3 cells in each group. Red fluorescence indicates JC-1 aggregates in healthy mitochondria, while green fluorescence represents JC-1 monomers in depolarized mitochondria. (I) Representative DCF-DA fluorescence images indicating intracellular ROS levels in bEnd.3 cells across all groups. (J) Quantification of JC-1 fluorescence ratio (green/red) representing mitochondrial membrane potential. (K) Quantification of DCF-DA fluorescence intensity reflecting ROS production. (L) Protein-protein interaction network for the top 10 up-regulated genes. (M) Protein–protein interaction network for the top 10 down-regulated genes. JC-1, 5,5’ ′,6,6′-tetrachloro-1,1′,3,3′-tetraethylbenzimidazolylcarbocyanine iodide; DCF-DA, 2′,7′-dichlorofluorescin diacetate, ∗ *p*-value <0.05, ∗∗∗ *p*-value <0.001.

To examine transcriptomic changes in ammonia-treated bEnd.3 cells following exendin-4 pretreatment, we performed total RNA sequencing and compared the ammonia plus exendin-4-treated bEnd.3 cell group with the ammonia-treated bEnd.3 cell group (Fig. 4). After controlling for multiple testing across 10,681 genes using the Benjamini–Hochberg false discovery rate (FDR), no genes reached transcriptome-wide significance at the FDR <0.05 threshold (n = 3 per group). Consequently, to identify potential biological trends for hypothesis generation, an exploratory screen was conducted based on an unadjusted p < 0.05, yielding 425 nominally significant candidates. The volcano plot (log2 fold change vs. –log10 p) highlights representative nominally significant candidates, and it is explicitly labeled as exploratory (Fig. 4A).

Among the nominally significant candidates, examples included Gm45783, MAP kinase activating death domain (Madd), Tafazzin, Trio, RNA binding motif protein 7 (Rbm7), peroxisomal biogenesis factor 11 beta (Pex11b), tetrapanin 5 (Tapan5), survival motor neuron 1 (Smn1), cystatin E/M (Cst6), phosphatidylethanolamine N-methyltransferase (Pemt), and H2B clustered histone 23 (H2bc23) (Fig. 4A). We further summarized the top 20 up-regulated genes, including H2bc23, Pemt, and Cst6 (Fig. 4B), and the top 20 down-regulated genes, including Gm45738, ataxin 7 (Atxn7), and Madd (Fig. 4C), ranked by fold change among exploratory candidates with an unadjusted p < 0.05 (Fig. 4B and C).

To validate the biological relevance of these exploratory candidates, quantitative real-time PCR (qRT-PCR) and Western blot analyses were performed. In agreement with the exploratory transcriptomic trends, Fig. 4D showed significantly increased mRNA levels of H2bc23, Fkbpl, and Pemt, alongside decreased mRNA levels of Pex11b, Tmem140, Madd, Stat2, and Oas3 in the ammonia plus exendin-4 treatment group (Fig. 4D). Furthermore, the protein level of HIF-1α was confirmed to be increased in the ammonia plus exendin-4 group compared to the ammonia-only group (Fig. 4E). Subsequently, exploratory GO analysis was performed on the top 400 nominal candidates (ranked by unadjusted p-value among those with p < 0.05). The biological trends suggested by the enriched GO terms included RNA processing, mRNA metabolic process, cellular response to stress, and mitochondrion (Fig. 4F). Additionally, exploratory KEGG pathway analysis nominated candidates involved in the HIF-1α signaling pathway, JAK-STAT signaling pathway, and lysosome (Fig. 4G). Consistent with the trends identified in the exploratory KEGG analysis, exendin-4 treatment was confirmed to increase HIF-1α protein levels in bEnd.3 cells under ammonia exposure (Fig. 4G). The high consistency between the exploratory transcriptomic profiling and independent validation of representative candidates across multiple regulatory pathways reinforces the reliability of our transcriptomic data.

JC-1 staining revealed a marked reduction in red fluorescence (JC-1 aggregates) and a concurrent increase in green fluorescence (JC-1 monomers) in ammonia-treated bEnd.3 cells, indicating substantial mitochondrial membrane potential loss. In contrast, exendin-4-treated cells exhibited a fluorescence pattern similar to that of the control group, with predominant red fluorescence. Cells exposed to ammonia with exendin-4 pretreatment showed partial restoration of red fluorescence and reduced green signal compared to that of the ammonia group, suggesting that exendin-4 mitigates ammonia-induced mitochondrial dysfunction (Fig. 4H). ROS production was evaluated using DCF-DA fluorescence (Figur e 4I). Quantification of the green/red fluorescence intensity ratio confirmed these findings. Ammonia-treated cells exhibited the highest green/red ratio, reflecting marked mitochondrial depolarization, whereas control and exendin-4 groups showed significantly lower ratios. Cells exposed to ammonia with exendin-4 pretreatment exhibited an intermediate ratio, indicating partial recovery of mitochondrial membrane potential (Fig. 4J). Ammonia exposure also caused a significant increase in green fluorescence intensity, consistent with elevated intracellular ROS levels (Fig. 4I). Conversely, exendin-4 treatment alone did not increase ROS production, with fluorescence levels comparable to those of the control. bEnd.3 cells treated with both ammonia and exendin-4 exhibited reduced DCF-DA fluorescence compared to those treated with ammonia alone, indicating that exendin-4 effectively attenuates ROS accumulation (Fig. 4I). Quantitative analysis of DCF-DA fluorescence intensity confirmed these findings, showing the highest ROS signal in ammonia-treated cells and a significant reduction in the ammonia plus exendin-4 group (Fig. 4K). Collectively, these imaging data indicate that exendin-4 treatment significantly restores mitochondrial membrane potential and reduces ROS generation in ammonia-exposed bEnd.3 cells. The protein-protein interaction networks among the top 10 increased and top 10 decreased genes was shown in Fig. 4L and M (Fig. 4L and M). In summary, exendin-4 induces exploratory transcriptomic changes in brain endothelial cells exposed to hyperammonic conditions.

## 4 Discussion

In this study, we demonstrated that BDL-induced hyperammonemia compromises BBB integrity, impairs synaptic plasticity and neurogenesis, and promotes neuroinflammation, ultimately contributing to cognitive decline in HE.

In the present study, we firstly confirmed the expression of brain dysfunction related markers in BDL mouse brain tissue. BDL surgery was employed as a representative model of chronic HE, which is characterized by liver fibrosis, systemic inflammation, and moderate hyperammonemia, rather than the acute and severe ammonia surges observed in toxin-induced HE models. To complement this, we applied 10 mM NH_4_Cl *in vitro* to mimic severe hyperammonemic stress, under which the protective effects of exendin-4 could still be evaluated. Taken together, our dual approach reflects both the chronic HE phenotype *in vivo* and the acute hyperammonemic condition *in vitro*, providing a broader understanding of BBB dysfunction and therapeutic efficacy. This study demonstrated that exendin-4 protects BBB integrity under hyperammonemic conditions. While the 10 mM NH_4_Cl concentration used *in vitro* is higher than typical serum ammonia levels observed in BDL mice or in patients with hepatic encephalopathy, it was intentionally chosen to represent a severe stress condition and to test whether the protective effect of exendin-4 persists under such stringent circumstances. This approach complements the BDL model, which reflects chronic hyperammonemia *in vivo,* by allowing us to probe acute and robust cellular responses *in vitro*. Future studies incorporating a dose–response design with lower, physiologically relevant concentrations will help to further refine the translational implications. Furthermore, while we utilized 10 nM of exendin-4—a well-established optimal concentration for *in vitro* GLP-1 receptor activation—we cannot rule out that higher doses might alter the magnitude of the protective response or potentially lead to receptor desensitization. Future studies encompassing a broader dose-response evaluation of exendin-4 will be necessary to determine the maximum therapeutic window and detailed dose-dependent mechanisms.

In the present study, we did not directly evaluate cell viability after exendin-4 exposure. However, accumulating evidence indicates that GLP-1 receptor agonists, including exendin-4, exert neuroprotective and cytoprotective effects rather than cytotoxicity. For example, exendin-4 has been reported to preserve blood–brain barrier integrity and attenuate oxidative stress in neuronal and endothelial models without reducing cell survival. Consistent with these reports, our data showed that exendin-4 treatment alone did not induce increases in ROS generation, disruption of tight junction proteins, or changes in pro-apoptotic markers, supporting the absence of cytotoxicity under our experimental conditions. Nevertheless, we acknowledge this as a limitation of the study, and future experiments will incorporate direct viability assays such as MTT or CCK-8 to confirm the safety profile of exendin-4 in our *in vitro* models.

In this study, we intentionally analyzed different sets of markers in astrocytes and endothelial cells to reflect their distinct biological roles in hyperammonemia. C8-D1a astrocytes were selected for cytokine and chemokine profiling, as astrocytes are a major source of inflammatory mediators and are crucial in orchestrating neuroinflammatory responses under pathological conditions. In contrast, bEnd.3 endothelial cells were examined for tight junction proteins (claudin-5, occludin) and VEGF, which are directly related to endothelial barrier integrity, angiogenesis, and BBB permeability. This approach allowed us to highlight the complementary contributions of astrocytic and endothelial responses in hepatic encephalopathy, rather than duplicating identical measurements across cell types.

An important limitation of this study is that Fig. 1A presents only a schematic illustration of the experimental design in the BDL mouse model, and does not provide direct morphological evidence. Although we provided morphological data through H&E staining of brain tissue (Fig. 3) and immunocytochemistry/confocal imaging of tight junction proteins in cultured endothelial and astrocytic cells (Supplementary Figures), direct histological confirmation from the mouse model was limited. Future studies will aim to incorporate more extensive morphological assessments *in vivo*, such as immunohistochemistry for BBB markers, to further validate the structural changes underlying hyperammonemia-induced BBB disruption.

The downregulation of tight junction proteins (occludin, claudin-5), synaptic markers (PSD95, SYP), and reduced DCX expression highlights a convergent mechanism in which barrier dysfunction, synaptic failure, and impaired neurogenesis exacerbate neurological deficits [10,[27], [28], [29], [30]]. These findings underscore the BBB as an active regulator of neural function in HE rather than a passive structural barrier. The observed regional disparity—significant in the cortex but trending in the hippocampus—highlights the complex nature of BBB disruption in HE. This may reflect region-specific sensitivity to ammonia toxicity, where cortical integrity is compromised more rapidly than hippocampal structures. While direct profiling of pro-inflammatory cytokines in BDL brain tissues was not performed in this study, the observed elevation of Cyp2e1 and reduced tight junction integrity strongly suggest an inflammatory milieu. Previous studies have consistently shown that hyperammonemia-induced ROS production triggers microglial activation and cytokine release, confirming the BDL model as a valid representative of neuroinflammation in HE [2,31]. The discrepancy between mRNA and protein levels in the hippocampus suggests that 2 weeks of BDL may be an early stage of pathology where transcriptional stress response occurs before substantial functional protein degradation is manifested. Furthermore, elevated GLP-1 receptor expression in the BDL brain may further reflect complex interactions with metabolic changes such as weight loss [32,33] or inflammation [34]. Specifically, this up-regulation may function as a compensatory mechanism, wherein the neurovascular unit increases receptor density to counteract hyperammonemia-induced oxidative stress and apoptosis. This “priming” of the GLP-1R signaling pathway could potentially enhance the brain's responsiveness to exogenous agonists like exendin-4, thereby facilitating the restoration of BBB integrity and synaptic markers as observed in our results. While the precise endogenous role of this increase requires further functional validation, it underscores the therapeutic potential of targeting the GLP-1R system in the chronic stages of HE. The study also showed that exendin-4 treatment protects against ammonia-induced BBB disruption. By preserving tight junction proteins, reducing pro-inflammatory cytokine production, and attenuating ROS generation and apoptosis in endothelial cells and astrocytes, exendin-4 supports barrier stability under hyperammonemic stress. These protective effects involve the modulation of mitochondrial function and the suppression of proinflammatory signaling. Specifically, GLP-1R activation by exendin-4 is known to stimulate cAMP/PKA and AMPK pathways, which in turn inhibit the nuclear translocation of NF-κB, a key transcriptional regulator of MMP-9. By suppressing the NF-κB/MMP-9 axis, exendin-4 prevents the enzymatic degradation of tight junction proteins such as claudin-5 and occludin. This is consistent with prior reports of GLP-1R/AMPK-mediated BBB protection in cerebrovascular disease [35], but extended here to the context of hyperammonemia-induced BBB disruption in HE. Furthermore, the observed reduction in ROS generation likely further stabilizes this pathway, as oxidative stress is a primary trigger for MMP-9 activation. Our *in vitro* BBB model further confirmed these effects: exendin-4 decreased permeability, preserved tight junction proteins, and suppressed MMP-9 expression under hyperammonemic conditions. In this study, 70 kDa FITC-dextran was utilized to evaluate macromolecular transport, reflecting the severe barrier disruption and protein leakage often observed in advanced HE. While smaller tracers might offer additional sensitivity to subtle junctional changes, our findings clearly demonstrate that exendin-4 provides significant protection against the passage of larger molecules, thereby stabilizing the neurovascular unit under severe hyperammonemic stress. Furthermore, we acknowledge that the present study did not include Transendothelial Electrical Resistance (TEER) measurements, which are widely considered a gold standard for evaluating real-time ionic permeability and subtle paracellular dysfunctions. However, the consistent correlation between the functional macromolecular leakage (FITC-dextran permeability) and the structural disruption of key tight junction proteins (claudin-5 and occludin) provides robust, cross-validated evidence of barrier compromise. Future studies incorporating TEER measurements, alongside smaller paracellular tracers, will be necessary to comprehensively profile the dynamic, real-time changes in BBB integrity under hyperammonemic stress. The reduction of inflammatory mediators, such as IP-10, KC/CXCL-1, M-CSF, and MCP-1, is particularly notable because these molecules are linked to BBB permeability, neuronal excitability, and neuroinflammatory cascades [[36], [37], [38], [39], [40], [41], [42], [43], [44]]. Together, these findings highlight exendin-4 as a potent regulator of neurovascular inflammation in HE. In the pathogenesis of hepatic encephalopathy, astrocytes are widely considered the primary target of ammonia toxicity due to their predominant role in ammonia detoxification via glutamine synthetase. Our data supports a hierarchical mechanism of BBB disruption: ammonia directly targets and stresses astrocytes, prompting them to act as active responders that secrete pro-inflammatory cytokines and MMP-9. These astrocyte-derived factors subsequently act as secondary effectors that degrade endothelial tight junctions, ultimately compromising BBB integrity. Therefore, the protective efficacy of exendin-4 lies not only in directly preserving endothelial cells but also in suppressing the primary astrocytic inflammatory response that drives barrier failure. Reduced ROS production indicated by decreased Cyp2e1 and Cyp4a1 mRNA levels [45] and suppressed apoptosis signaling—reflected by lower Naip mRNA level [46] were observed in ammonia-exposed bEnd.3 cells and astrocytes. A decrease in mitochondrial DNA regulatory gene Tfam [47] and the electron transport chain gene Cox7a1 further suggests that exendin-4 may enhance mitochondrial function in BBB component cells despite ammonia-induced toxicity. Moreover, reduced IL-6 [48] and increased IL-10 [49] expression confirm its anti-inflammatory effects in endothelial cells under hyperammonemic stress.

Transcriptomic profiling provided additional mechanistic insight. Upregulated genes such as Pex11b and Fkbpl implicate endothelial peroxisomal stress and STAT3 signaling [[50], [51], [52], [53], [54]], while downregulated genes, including Ptp4a1, STAT2, and Tmem140, suggest reduced endothelial inflammation, immune modulation, and oncogenic signaling [[55], [56], [57], [58]]. The transcriptomic findings in this study should be interpreted as exploratory, as the small sample size precluded genes from surviving strict FDR correction. While the RNA-sequencing data alone lacks sufficient statistical power for definitive conclusions, it successfully served as a hypothesis-generating tool. Importantly, our confidence in the specific molecular pathways highlighted by this screen is firmly grounded in our orthogonal validations. The consistent verification of representative up- and down-regulated genes via independent RT-PCR, alongside the protein-level confirmation of HIF-1α, robustly substantiates the biological reliability of these top candidates in mediating the protective effects of exendin-4. Enriched GO and KEGG pathways suggest potential biological trends such as RNA processing, mitochondrial function, and HIF-1α/JAK-STAT signaling further indicating that exendin-4 may contribute to enhancing endothelial resilience under hyperammonemic stress [[59], [60], [61], [62], [63], [64], [65], [66], [67], [68], [69], [70], [71]]. Furthermore, increased astrocytic HIF-1α expression under exendin-4 treatment was associated with reduced BBB permeability and attenuated inflammation [[72], [73], [74]]. The restoration of mitochondrial membrane potential and reduced ROS generation are closely aligned with the up-regulation of mitochondrial regulatory genes such as *Tfam* and *Cox7a1*. Although the direct causal link between the exploratory transcriptomic shifts and functional outcomes remains to be definitively proven, the consistency across our molecular and functional assays strongly suggests that exendin-4 stabilizes the BBB by enhancing mitochondrial resilience. From a translational perspective, these findings have significant clinical implications. HE currently lacks effective therapies beyond ammonia-lowering strategies. The transcriptomic findings in this study should be interpreted as exploratory, as the small sample size precluded genes from surviving strict FDR correction. However, the biological relevance of these nominal candidates was supported by subsequent RT-PCR validation and functional assays. Our findings suggest that GLP-1 receptor agonists already widely prescribed for diabetes and obesity could be repurposed to target neurovascular dysfunction in HE. By stabilizing the BBB, suppressing neuroinflammation, and supporting mitochondrial function, exendin-4 emerges as a promising multipronged therapeutic candidate. Therefore, future studies should evaluate its efficacy and safety in patients with HE, as this strategy may provide a clinically actionable approach to improve neurological outcomes in this debilitating condition.

## 5 Conclusions

In conclusion, we propose that GLP-1 and exendin-4 attenuate inflammation, apoptosis, ROS generation, mitochondrial dysfunction, pro-inflammatory cytokine secretion, tight junction protein loss, and increased BBB permeability, thereby protecting against BBB breakdown in HE. This study underscores the therapeutic potential of GLP-1 for neuropathologies associated with BBB disruption in patients with HE. While limited by the absence of mechanistic data on exendin-4–mediated regulation of BBB permeability, this study is significant as the first to demonstrate the BBB-protective effects of exendin-4 in a model of HE. Based on these findings, further animal and clinical studies are warranted to elucidate the specific therapeutic mechanisms of GLP-1 and to advance the development of interventions targeting BBB disruption in HE.

## Fundings

This research was supported by the National Research Foundation of Korea (NRF) grant funded by the Korean government (Grant No. RS-2025-02213506, RS-2025-19612989)(Juhyun Song). This research was supported by a grant from the Korea Health Technology R&D Project through the Korea Health Industry Development Institute (KHIDI), funded by the Ministry of Health & Welfare, Republic of Korea (Grant number: RS-2025-19252970) (Juhyun Song).

## CRediT authorship contribution statement

**Seo Yeon Ahn:** Conceptualization, Data curation, Formal analysis, Investigation, Methodology, Software, Validation, Visualization, Writing – original draft. **Danbi Jo:** Data curation, Formal analysis, Investigation, Methodology, Validation, Visualization. **Seo Yoon Choi:** Data curation, Formal analysis, Investigation, Methodology. **Juhyun Song:** Conceptualization, Data curation, Formal analysis, Funding acquisition, Investigation, Methodology, Project administration, Resources, Software, Supervision, Validation, Visualization, Writing – original draft, Writing – review & editing.

## Declaration of competing interest

The authors declare no conflicts of interest.

## Data Availability

All data presented in this study are included in the article. Additional data supporting the findings are available from the corresponding author upon reasonable request.

## References

[1] Vilstrup H., Amodio P., Bajaj J., Cordoba J., Ferenci P., Mullen K.D., Weissenborn K., Wong P. (2014). Hepatic encephalopathy in chronic liver disease: 2014 practice guideline by the American association for the study of liver diseases and the European association for the study of the liver. Hepatology.

[2] Felipo V. (2013). Hepatic encephalopathy: effects of liver failure on brain function. Nat. Rev. Neurosci..

[3] Haussinger D., Gorg B. (2010). Interaction of oxidative stress, astrocyte swelling and cerebral ammonia toxicity. Curr. Opin. Clin. Nutr. Metab. Care.

[4] Romero-Gomez M., Montagnese S., Jalan R. (2015). Hepatic encephalopathy in patients with acute decompensation of cirrhosis and acute-on-chronic liver failure. J. Hepatol..

[5] Weissenborn K., Ahl B., Fischer-Wasels D., van den Hoff J., Hecker H., Burchert W., Kostler H. (2007). Correlations between magnetic resonance spectroscopy alterations and cerebral ammonia and glucose metabolism in cirrhotic patients with and without hepatic encephalopathy. Gut.

[6] Capocaccia L., Angelico M. (1991). Fulminant hepatic failure. Clinical features, etiology, epidemiology, and current management. Dig. Dis. Sci..

[7] Chepkova A.N., Sergeeva O.A., Gorg B., Haas H.L., Klocker N., Haussinger D. (2017). Impaired novelty acquisition and synaptic plasticity in congenital hyperammonemia caused by hepatic glutamine synthetase deficiency. Sci. Rep..

[8] Dhanda S., Sandhir R. (2018). Blood-brain barrier permeability is exacerbated in experimental model of hepatic encephalopathy via MMP-9 activation and downregulation of tight junction proteins. Mol. Neurobiol..

[9] Jayakumar A.R., Norenberg M.D. (2018). Hyperammonemia in hepatic encephalopathy. J. Clin. Exp. Hepatol..

[10] Hawkins B.T., Davis T.P. (2005). The blood-brain barrier/neurovascular unit in health and disease. Pharmacol. Rev..

[11] Ding X., Saxena N.K., Lin S., Gupta N.A., Anania F.A. (2006). Exendin-4, a glucagon-like protein-1 (GLP-1) receptor agonist, reverses hepatic steatosis in ob/ob mice. Hepatology.

[12] Egan J.M., Clocquet A.R., Elahi D. (2002). The insulinotropic effect of acute exendin-4 administered to humans: comparison of nondiabetic state to type 2 diabetes. J. Clin. Endocrinol. Metab..

[13] Kolterman O.G., Buse J.B., Fineman M.S., Gaines E., Heintz S., Bicsak T.A., Taylor K., Kim D., Aisporna M., Wang Y., Baron A.D. (2003). Synthetic exendin-4 (exenatide) significantly reduces postprandial and fasting plasma glucose in subjects with type 2 diabetes. J. Clin. Endocrinol. Metab..

[14] Szayna M., Doyle M.E., Betkey J.A., Holloway H.W., Spencer R.G., Greig N.H., Egan J.M. (2000). Exendin-4 decelerates food intake, weight gain, and fat deposition in Zucker rats. Endocrinology.

[15] Yabut J.M., Drucker D.J. (2023). Glucagon-like Peptide-1 receptor-based therapeutics for metabolic liver disease. Endocr. Rev..

[16] Yen F.S., Hou M.C., Cheng-Chung Wei J., Shih Y.H., Hsu C.Y., Hsu C.C., Hwu C.M. (2024). Glucagon-like Peptide-1 receptor agonist use in patients with liver cirrhosis and type 2 diabetes. Clin. Gastroenterol. Hepatol..

[17] Teramoto S., Miyamoto N., Yatomi K., Tanaka Y., Oishi H., Arai H., Hattori N., Urabe T. (2011). Exendin-4, a glucagon-like peptide-1 receptor agonist, provides neuroprotection in mice transient focal cerebral ischemia. J. Cereb. Blood Flow Metab..

[18] Yoon G., Kim Y.K., Song J. (2020). Glucagon-like peptide-1 suppresses neuroinflammation and improves neural structure. Pharmacol. Res..

[19] Jo D., Yoon G., Song J. (2021). Role of Exendin-4 in brain insulin resistance, mitochondrial function, and neurite outgrowth in neurons under palmitic acid-induced oxidative stress. Antioxidants (Basel).

[20] Wang M., Yoon G., Song J., Jo J. (2021). Exendin-4 improves long-term potentiation and neuronal dendritic growth in vivo and in vitro obesity condition. Sci. Rep..

[21] Fukuda S., Nakagawa S., Tatsumi R., Morofuji Y., Takeshita T., Hayashi K., Tanaka K., Matsuo T., Niwa M. (2016). Glucagon-like Peptide-1 strengthens the barrier integrity in primary cultures of rat brain endothelial cells under basal and hyperglycemia conditions. J. Mol. Neurosci..

[22] Shan Y., Tan S., Lin Y., Liao S., Zhang B., Chen X., Wang J., Deng Z., Zeng Q., Zhang L., Wang Y., Hu X., Qiu W., Peng L., Lu Z. (2019). The glucagon-like peptide-1 receptor agonist reduces inflammation and blood-brain barrier breakdown in an astrocyte-dependent manner in experimental stroke. J. Neuroinflammation.

[23] Back A., Tupper K.Y., Bai T., Chiranand P., Goldenberg F.D., Frank J.I., Brorson J.R. (2011). Ammonia-induced brain swelling and neurotoxicity in an organotypic slice model. Neurol. Res..

[24] Ismail F.S., Faustmann T.J., Corvace F., Tsvetanova A., Moinfar Z., Faustmann P.M. (2021). Ammonia induced microglia activation was associated with limited effects on connexin 43 and aquaporin 4 expression in an astrocyte-microglia co-culture model. BMC Neurosci..

[25] Bray N.L., Pimentel H., Melsted P., Pachter L. (2016). Near-optimal probabilistic RNA-seq quantification. Nat. Biotechnol..

[26] Liberzon A., Subramanian A., Pinchback R., Thorvaldsdottir H., Tamayo P., Mesirov J.P. (2011). Molecular signatures database (MSigDB) 3.0. Bioinformatics.

[27] Balthazart J., Ball G.F. (2014). Doublecortin is a highly valuable endogenous marker of adult neurogenesis in canaries. Commentary on Vellema M et al. (2014): evaluating the predictive value of doublecortin as a marker for adult neurogenesis in canaries (Serinus canaria). Brain Behav. Evol..

[28] Chen F., Ohashi N., Li W., Eckman C., Nguyen J.H. (2009). Disruptions of occludin and claudin-5 in brain endothelial cells in vitro and in brains of mice with acute liver failure. Hepatology.

[29] McMillin M.A., Frampton G.A., Seiwell A.P., Patel N.S., Jacobs A.N., DeMorrow S. (2015). TGFbeta1 exacerbates blood-brain barrier permeability in a mouse model of hepatic encephalopathy via upregulation of MMP9 and downregulation of claudin-5. Lab. Invest..

[30] Monfort P., Cauli O., Montoliu C., Rodrigo R., Llansola M., Piedrafita B., El Mlili N., Boix J., Agusti A., Felipo V. (2009). Mechanisms of cognitive alterations in hyperammonemia and hepatic encephalopathy: therapeutical implications. Neurochem. Int..

[31] Jayakumar A.R., Norenberg M.D. (2018). Aberrant brain bile acid signaling and cholesterol accumulation: a new look at mechanisms in hepatic encephalopathy. Cell. Mol. Gastroenterol. Hepatol..

[32] Kanoski S.E., Hayes M.R., Skibicka K.P. (2016). GLP-1 and weight loss: unraveling the diverse neural circuitry. Am. J. Physiol. Regul. Integr. Comp. Physiol..

[33] Moiz A., Filion K.B., Tsoukas M.A., Yu O.H., Peters T.M., Eisenberg M.J. (2025). Mechanisms of GLP-1 receptor agonist-induced weight loss: a review of central and peripheral pathways in appetite and energy regulation. Am. J. Med..

[34] Sato T., Shimizu T., Fujita H., Imai Y., Drucker D.J., Seino Y., Yamada Y. (2020). GLP-1 receptor signaling differentially modifies the outcomes of sterile vs viral pulmonary inflammation in Male mice. Endocrinology.

[35] Xie Z., Enkhjargal B., Nathanael M., Wu L., Zhu Q., Zhang T., Tang J., Zhang J.H. (2021). Exendin-4 preserves blood-brain barrier integrity via glucagon-like peptide 1 receptor/activated protein kinase-dependent nuclear factor-kappa B/Matrix Metalloproteinase-9 inhibition after subarachnoid hemorrhage in rat. Front. Mol. Neurosci..

[36] Andjelkovic A.V., Kerkovich D., Pachter J.S. (2000). Monocyte:astrocyte interactions regulate MCP-1 expression in both cell types. J. Leukoc. Biol..

[37] Burkhart A., Helgudottir S.S., Mahamed Y.A., Fruergaard M.B., Holm-Jacobsen J.N., Haraldsdottir H., Dahl S.E., Pretzmann F., Routhe L.G., Lambertsen K., Moos T., Thomsen M.S. (2024). Activation of glial cells induces proinflammatory properties in brain capillary endothelial cells in vitro. Sci. Rep..

[38] Dong F., Du Y.R., Xie W., Strong J.A., He X.J., Zhang J.M. (2012). Increased function of the TRPV1 channel in small sensory neurons after local inflammation or in vitro exposure to the pro-inflammatory cytokine GRO/KC. Neurosci. Bull..

[39] Hamilton T.A., Zhao C., Pavicic P.G., Datta S. (2014). Myeloid colony-stimulating factors as regulators of macrophage polarization. Front. Immunol..

[40] Li F., Wang Y., Yu L., Cao S., Wang K., Yuan J., Wang C., Wang K., Cui M., Fu Z.F. (2015). Viral infection of the central nervous system and neuroinflammation precede blood-brain barrier disruption during Japanese encephalitis virus infection. J. Virol..

[41] Thompson W.L., Karpus W.J., Van Eldik L.J. (2008). MCP-1-deficient mice show reduced neuroinflammatory responses and increased peripheral inflammatory responses to peripheral endotoxin insult. J. Neuroinflammation.

[42] Wang J.G., Strong J.A., Xie W., Yang R.H., Coyle D.E., Wick D.M., Dorsey E.D., Zhang J.M. (2008). The chemokine CXCL1/growth related oncogene increases sodium currents and neuronal excitability in small diameter sensory neurons. Mol. Pain.

[43] Wang K., Wang H., Lou W., Ma L., Li Y., Zhang N., Wang C., Li F., Awais M., Cao S., She R., Fu Z.F., Cui M. (2018). IP-10 promotes blood-brain barrier damage by inducing tumor necrosis factor alpha production in Japanese encephalitis. Front. Immunol..

[44] Zhang Z.J., Cao D.L., Zhang X., Ji R.R., Gao Y.J. (2013). Chemokine contribution to neuropathic pain: respective induction of CXCL1 and CXCR2 in spinal cord astrocytes and neurons. Pain.

[45] Schattenberg J.M., Czaja M.J. (2014). Regulation of the effects of CYP2E1-induced oxidative stress by JNK signaling. Redox Biol..

[46] Diez E., Yaraghi Z., MacKenzie A., Gros P. (2000). The neuronal apoptosis inhibitory protein (Naip) is expressed in macrophages and is modulated after phagocytosis and during intracellular infection with Legionella pneumophila. J. Immunol..

[47] Fiebig C., Keiner S., Ebert B., Schaffner I., Jagasia R., Lie D.C., Beckervordersandforth R. (2019). Mitochondrial dysfunction in astrocytes impairs the generation of reactive astrocytes and enhances neuronal cell death in the cortex upon photothrombotic lesion. Front. Mol. Neurosci..

[48] Luo M., Li L., Yang E.N., Dai C.Y., Liang S.R., Cao W.K. (2013). Correlation between interleukin-6 and ammonia in patients with overt hepatic encephalopathy due to cirrhosis. Clin. Res. Hepatol. Gastroenterol..

[49] Santos R.P.C., Toscano E.C.B., Rachid M.A. (2023). Anti-inflammatory strategies for hepatic encephalopathy: preclinical studies. Arq. Neuropsiquiatr..

[50] Annett S., Moore G., Short A., Marshall A., McCrudden C., Yakkundi A., Das S., McCluggage W.G., Nelson L., Harley I., Moustafa N., Kennedy C.J., deFazio A., Brand A., Sharma R., Brennan D., O'Toole S., O'Leary J., Bates M., O'Riain C., O'Connor D., Furlong F., McCarthy H., Kissenpfennig A., McClements L., Robson T. (2020). FKBPL-based peptide, ALM201, targets angiogenesis and cancer stem cells in ovarian cancer. Br. J. Cancer.

[51] Koch J., Brocard C. (2011). Membrane elongation factors in organelle maintenance: the case of peroxisome proliferation. Biomol. Concepts.

[52] McKeen H.D., McAlpine K., Valentine A., Quinn D.J., McClelland K., Byrne C., O'Rourke M., Young S., Scott C.J., McCarthy H.O., Hirst D.G., Robson T. (2008). A novel FK506-like binding protein interacts with the glucocorticoid receptor and regulates steroid receptor signaling. Endocrinology.

[53] Vasko R., Ratliff B.B., Bohr S., Nadel E., Chen J., Xavier S., Chander P., Goligorsky M.S. (2013). Endothelial peroxisomal dysfunction and impaired pexophagy promotes oxidative damage in lipopolysaccharide-induced acute kidney injury. Antioxidants Redox Signal..

[54] Yakkundi A., McCallum L., O'Kane A., Dyer H., Worthington J., McKeen H.D., McClements L., Elliott C., McCarthy H.O., Hirst D.G., Robson T. (2013). The anti-migratory effects of FKBPL and its peptide derivative, AD-01: regulation of CD44 and the cytoskeletal pathway. PLoS One.

[55] Cho M.J., Lee D.G., Lee J.W., Hwang B., Yoon S.J., Lee S.J., Park Y.J., Park S.H., Lee H.G., Kim Y.H., Lee C.H., Lee J., Lee N.K., Han T.S., Cho H.S., Moon J.H., Lee G.S., Bae K.H., Hwang G.S., Lee S.H., Chung S.J., Shim S., Cho J., Oh G.T., Kwon Y.G., Park J.G., Min J.K. (2023). Endothelial PTP4A1 mitigates vascular inflammation via USF1/A20 axis-mediated NF-kappaB inactivation. Cardiovasc. Res..

[56] Lee C.J., An H.J., Cho E.S., Kang H.C., Lee J.Y., Lee H.S., Cho Y.Y. (2020). Stat2 stability regulation: an intersection between immunity and carcinogenesis. Exp. Mol. Med..

[57] Li B., Huang M.Z., Wang X.Q., Tao B.B., Zhong J., Wang X.H., Zhang W.C., Li S.T. (2015). TMEM140 is associated with the prognosis of glioma by promoting cell viability and invasion. J. Hematol. Oncol..

[58] Schutze K., Gross M., Cornils K., Wustrau K., Schneppenheim S., Lenhartz H., Korenke G.C., Janka G., Ledig S., Muller I., Ehl S., Lehmberg K. (2023). MAP kinase activating death domain deficiency is a novel cause of impaired lymphocyte cytotoxicity. Blood Adv..

[59] Biamonti G., Caceres J.F. (2009). Cellular stress and RNA splicing. Trends Biochem. Sci..

[60] Carew N.T., Nelson A.M., Liang Z., Smith S.M., Milcarek C. (2018). Linking endoplasmic reticular stress and alternative splicing. Int. J. Mol. Sci..

[61] Digiovanni S., Lorenzati M., Bianciotto O.T., Godel M., Fontana S., Akman M., Costamagna C., Couraud P.O., Buffo A., Kopecka J., Riganti C., Salaroglio I.C. (2024). Blood-brain barrier permeability increases with the differentiation of glioblastoma cells in vitro. Fluids Barriers CNS.

[62] Doll D.N., Hu H., Sun J., Lewis S.E., Simpkins J.W., Ren X. (2015). Mitochondrial crisis in cerebrovascular endothelial cells opens the blood-brain barrier. Stroke.

[63] Dubois L.G., Campanati L., Righy C., D'Andrea-Meira I., Spohr T.C., Porto-Carreiro I., Pereira C.M., Balca-Silva J., Kahn S.A., DosSantos M.F., Oliveira Mde A., Ximenes-da-Silva A., Lopes M.C., Faveret E., Gasparetto E.L., Moura-Neto V. (2014). Gliomas and the vascular fragility of the blood brain barrier. Front. Cell. Neurosci..

[64] Jain M., Singh M.K., Shyam H., Mishra A., Kumar S., Kumar A., Kushwaha J. (2021). Role of JAK/STAT in the neuroinflammation and its association with neurological disorders. Ann. Neurosci..

[65] Laili I.N., Nasir M.H.M., Jufri N.F., Ibrahim F.W., Hamid A. (2023). Lysosomal dysfunction induced cytosolic vacuolation and increased intracellular amyloid-beta 42 (Abeta42) in human brain endothelial cells (HBEC-5i). Biomed. Pharmacother..

[66] Pifferi F., Laurent B., Plourde M. (2021). Lipid transport and metabolism at the blood-brain interface: implications in health and disease. Front. Physiol..

[67] Shen Y., Gu J., Liu Z., Xu C., Qian S., Zhang X., Zhou B., Guan Q., Sun Y., Wang Y., Jin X. (2018). Inhibition of HIF-1alpha reduced blood brain barrier damage by regulating MMP-2 and VEGF during acute cerebral ischemia. Front. Cell. Neurosci..

[68] Tang Y.Y., Wang D.C., Wang Y.Q., Huang A.F., Xu W.D. (2022). Emerging role of hypoxia-inducible factor-1alpha in inflammatory autoimmune diseases: a comprehensive review. Front. Immunol..

[69] Tsai C.Y., Wu J.C.C., Wu C.J., Chan S.H.H. (2022). Protective role of VEGF/VEGFR2 signaling against high fatality associated with hepatic encephalopathy via sustaining mitochondrial bioenergetics functions. J. Biomed. Sci..

[70] Wang Y., Wu J., Wang J., He L., Lai H., Zhang T., Wang X., Li W. (2023). Mitochondrial oxidative stress in brain microvascular endothelial cells: triggering blood-brain barrier disruption. Mitochondrion.

[71] Yang J.L., Mukda S., Chen S.D. (2018). Diverse roles of mitochondria in ischemic stroke. Redox Biol..

[72] Badawi Y., Ramamoorthy P., Shi H. (2012). Hypoxia-inducible factor 1 protects hypoxic astrocytes against glutamate toxicity. ASN Neuro.

[73] Baumann J., Tsao C.C., Huang S.F., Gassmann M., Ogunshola O.O. (2021). Astrocyte-specific hypoxia-inducible factor 1 (HIF-1) does not disrupt the endothelial barrier during hypoxia in vitro. Fluids Barriers CNS.

[74] Jeon G.W., Sheldon R.A., Ferriero D.M. (2019). Hypoxia-inducible factor: role in cell survival in superoxide dismutase overexpressing mice after neonatal hypoxia-ischemia. Korean J. Pediatr..

