# Supplementary material for: Exendin-4 protects brain endothelial cell damage against hyperammonemic condition
Source: Biochem Biophys Rep. 2026 May 22;46:102644. doi: 10.1016/j.bbrep.2026.102644 (PMC13217415; doi:10.1016/j.bbrep.2026.102644)
Supplement: Multimedia component 1 [file mmc1.pdf]

Figure 2D

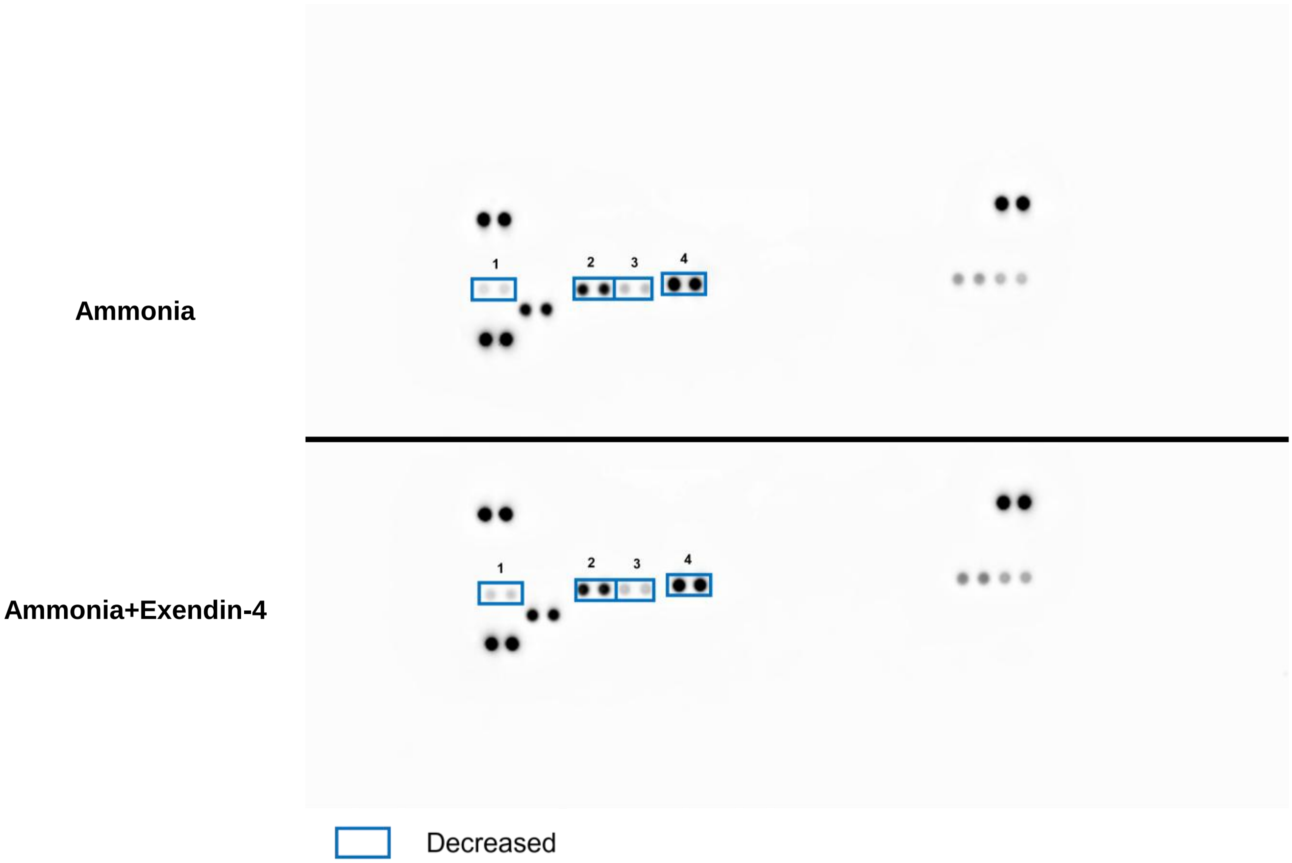

Ammonia
Ammonia+Exendin-4
1
2
3
4
Decreased

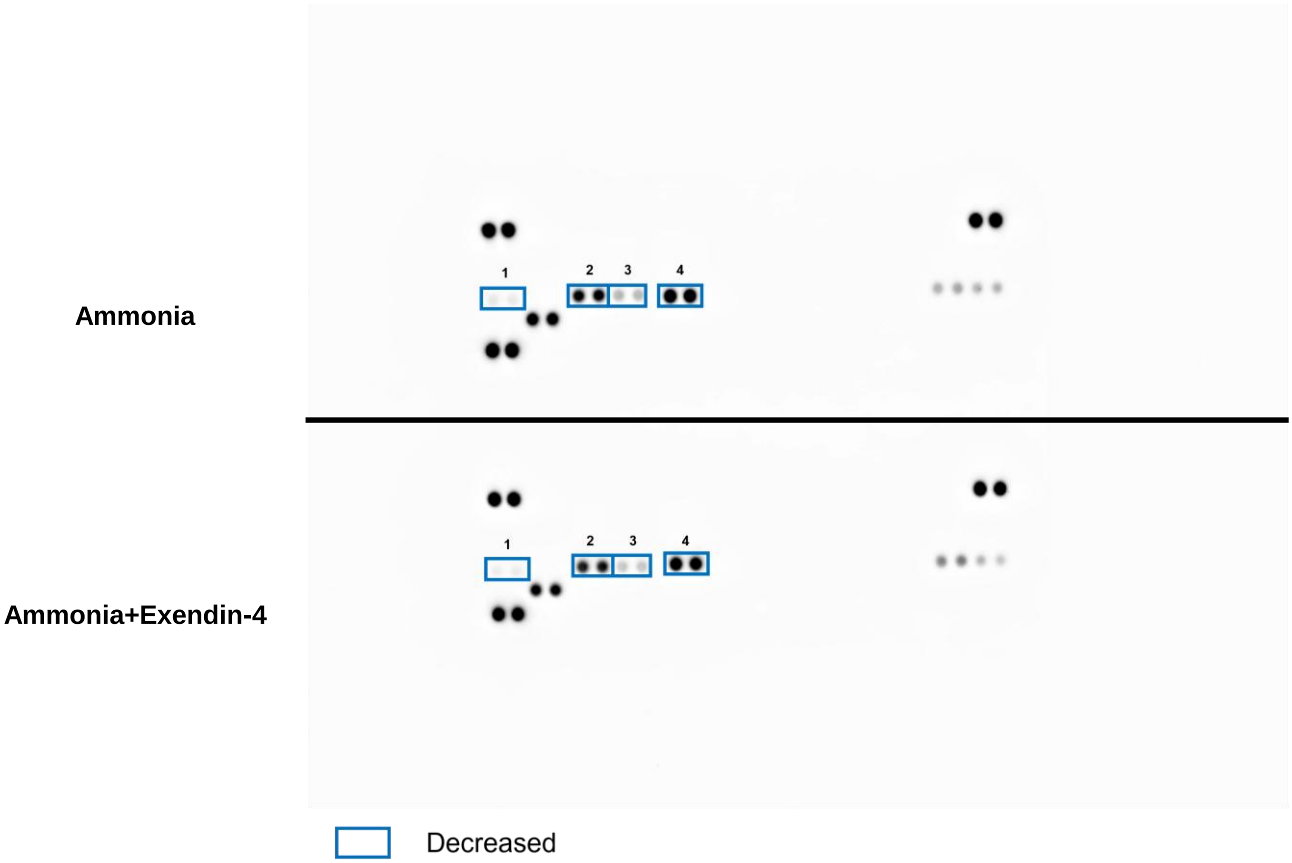

Ammonia
Ammonia+Exendin-4
1
2
3
4
Decreased

Figure 2G,H: Virtual bEnd.3 cell image in all groups

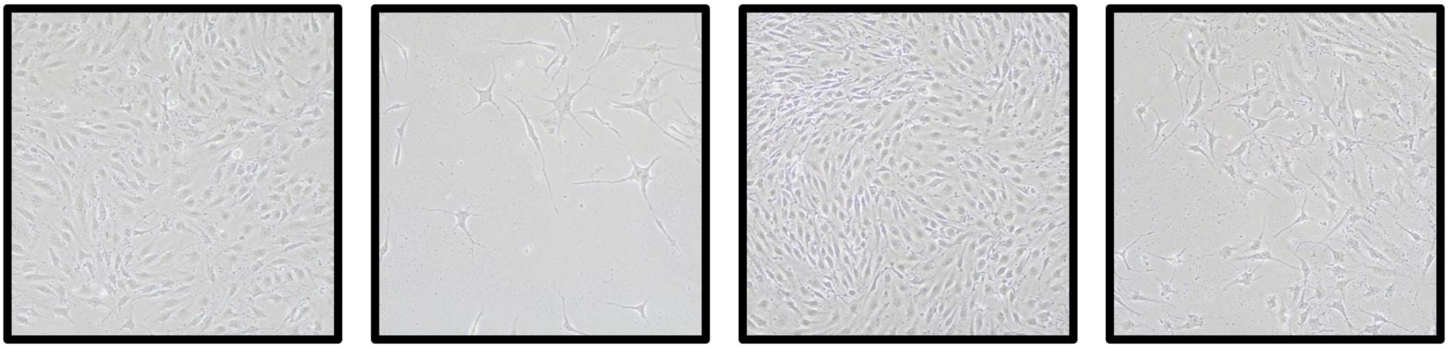

Ctr | Ammonia | Exendin-4 | Ammonia+Exendin-4

Figure 4H: Virtual bEnd.3 cell image in all groups

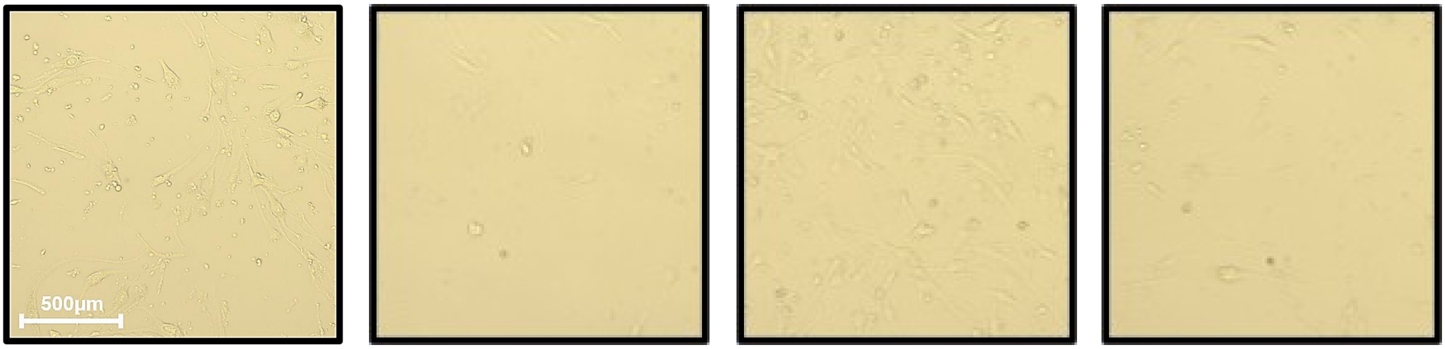

Ctr | Ammonia | Exendin-4 | Ammonia+Exendin-4

Figure 4I: Virtual bEnd.3 cell image in all groups

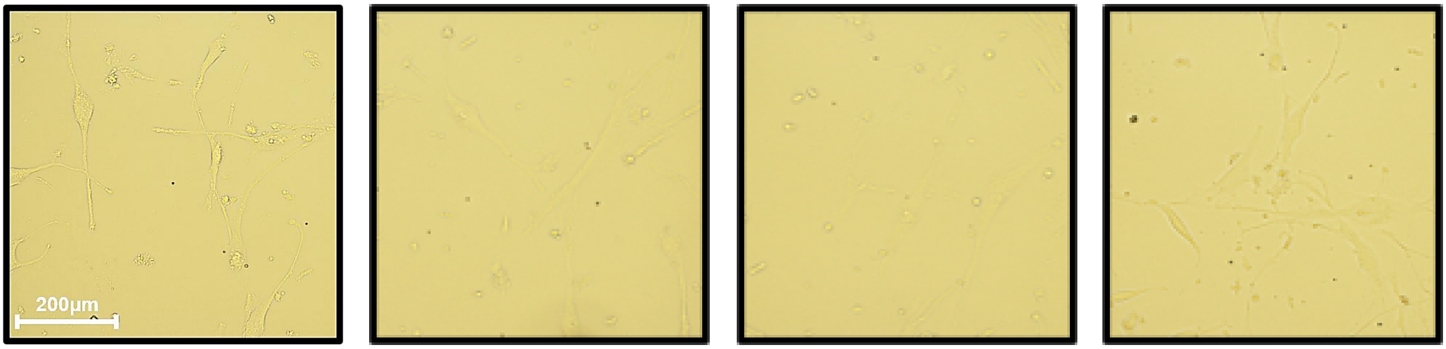

Ctr | Ammonia | Exendin-4 | Ammonia+Exendin-4
