# Supplementary material for: Exendin-4 protects brain endothelial cell damage against hyperammonemic condition
Source: Biochem Biophys Rep. 2026 May 22;46:102644. doi: 10.1016/j.bbrep.2026.102644 (PMC13217415; doi:10.1016/j.bbrep.2026.102644)
Supplement: Multimedia component 2 [file mmc2.pdf]

Figure 1D

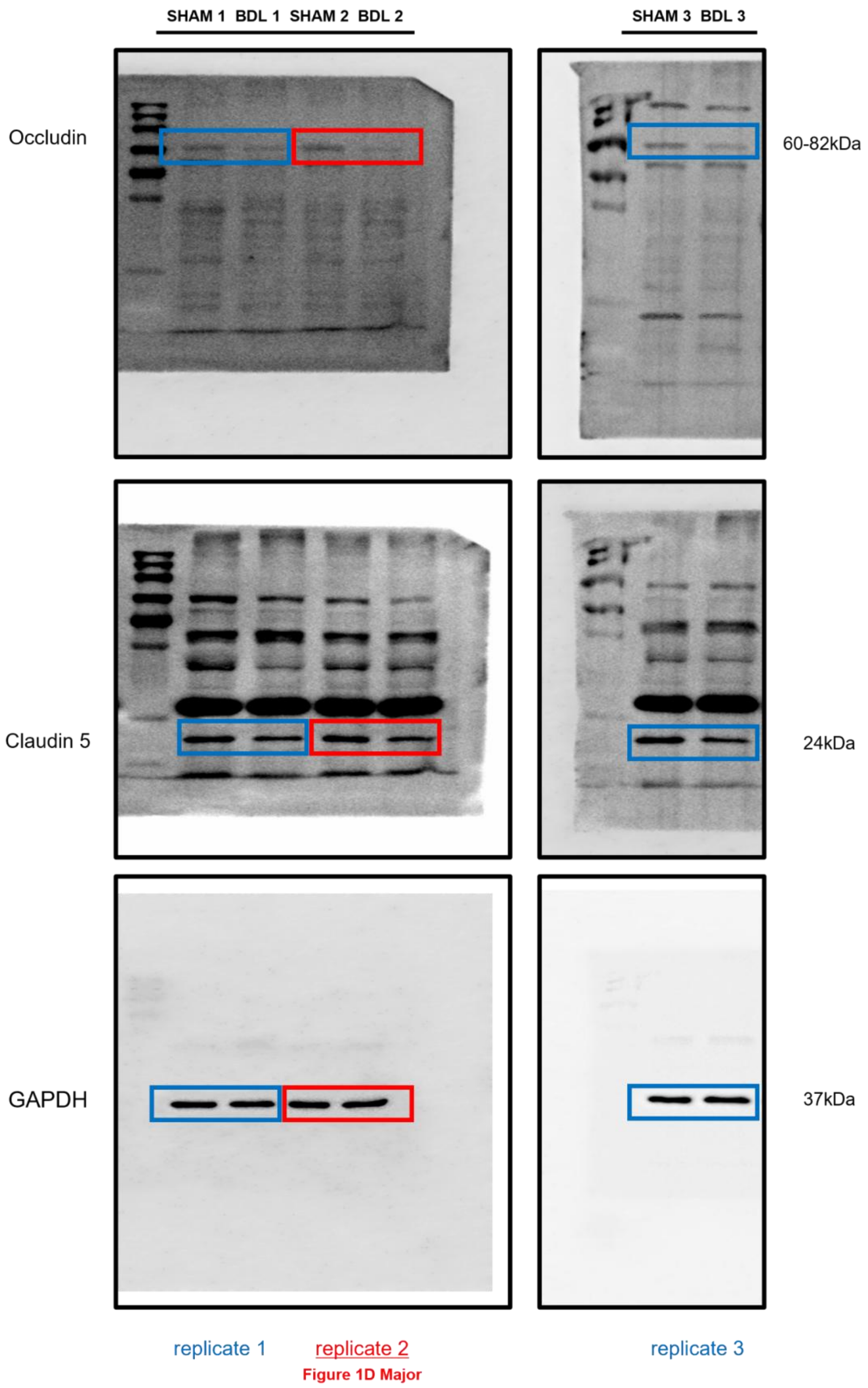

SHAM 1 BDL 1 SHAM 2 BDL 2
SHAM 3 BDL 3
Occludin
60-82kDa
Claudin 5
24kDa
GAPDH
37kDa
replicate 1
replicate 2
Figure 1D Major
replicate 3

Figure 1E

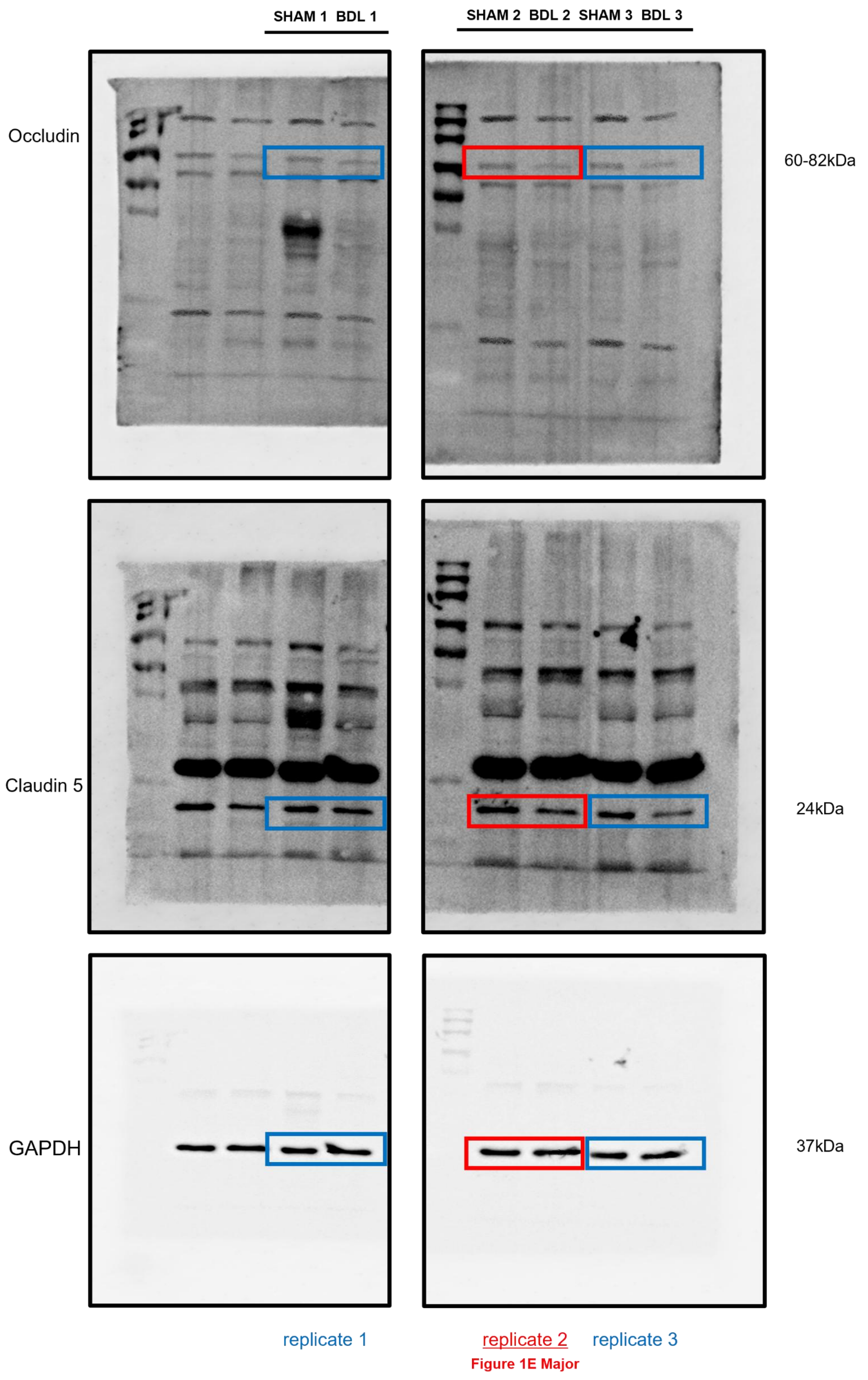
SHAM 1 BDL 1
SHAM 2 BDL 2 SHAM 3 BDL 3
Occludin
60-82kDa
Claudin 5
24kDa
GAPDH
37kDa
replicate 1
replicate 2
replicate 3
Figure 1E Major

Figure 1F

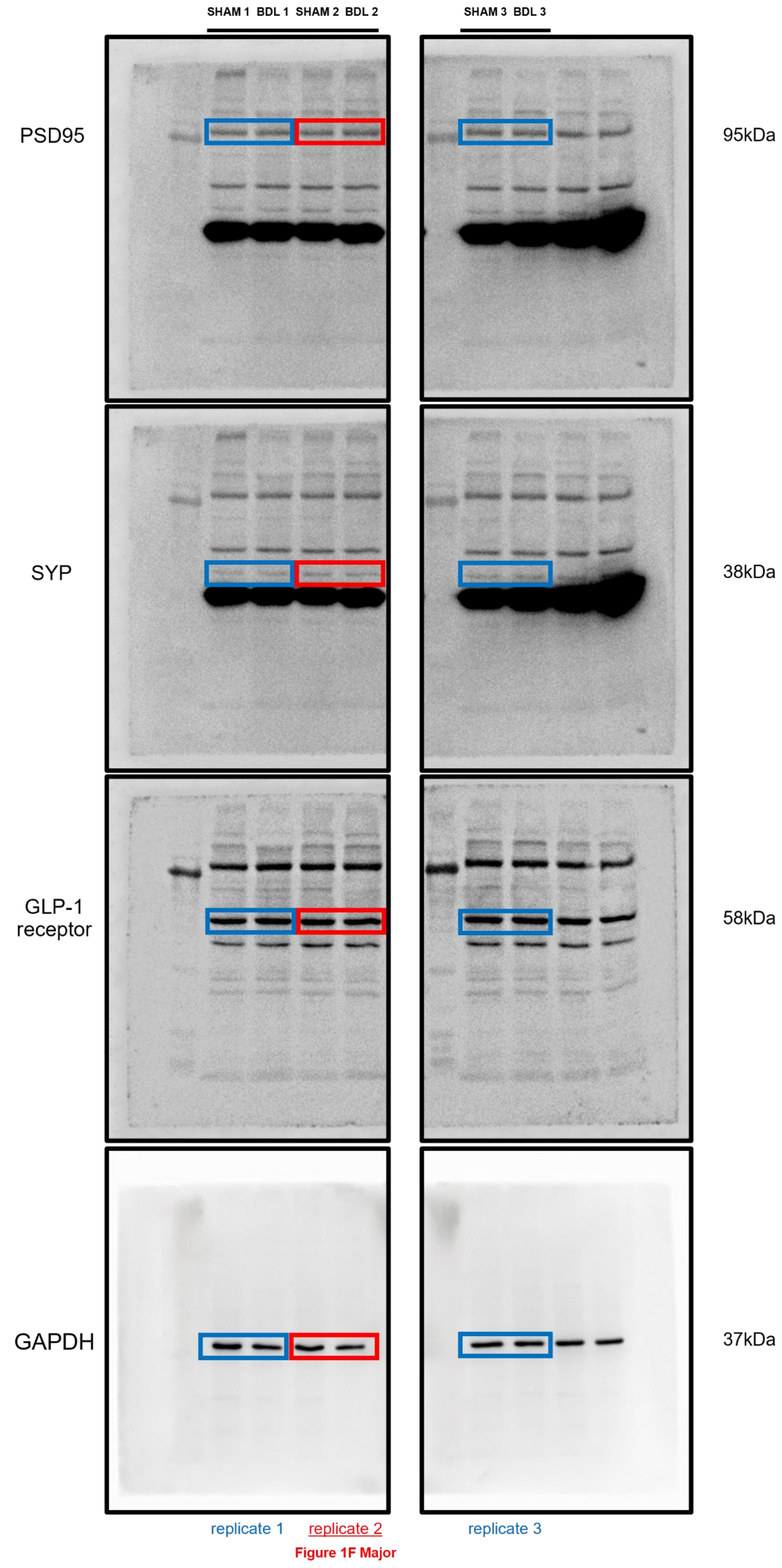
SHAM 1
BDL 1
SHAM 2
BDL 2
SHAM 3
BDL 3
PSD95
95kDa
SYP
38kDa
GLP-1 receptor
58kDa
GAPDH
37kDa
replicate 1
replicate 2
replicate 3
Figure 1F Major

Figure 1F

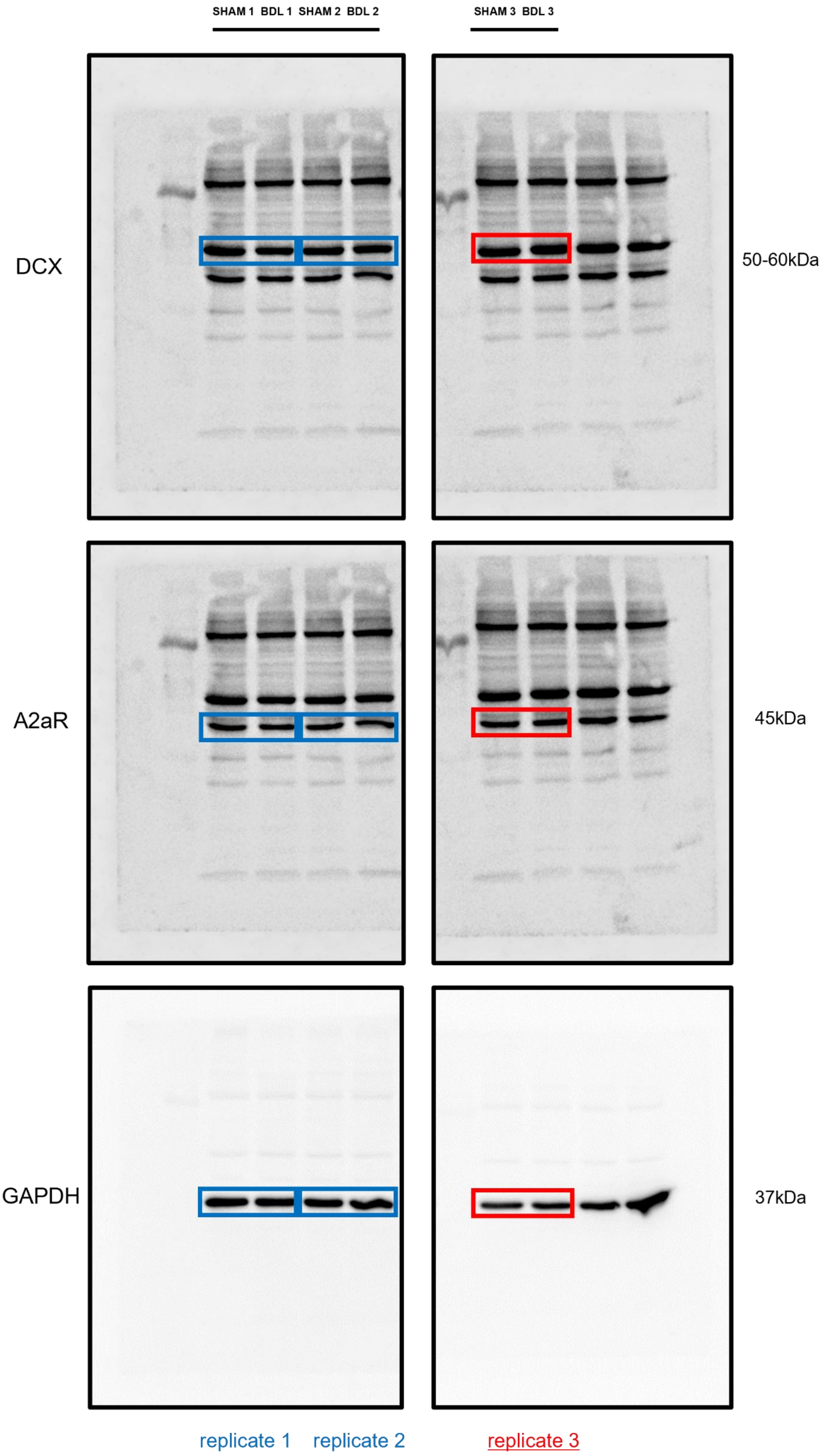

Figure 1F Major

Figure 1G

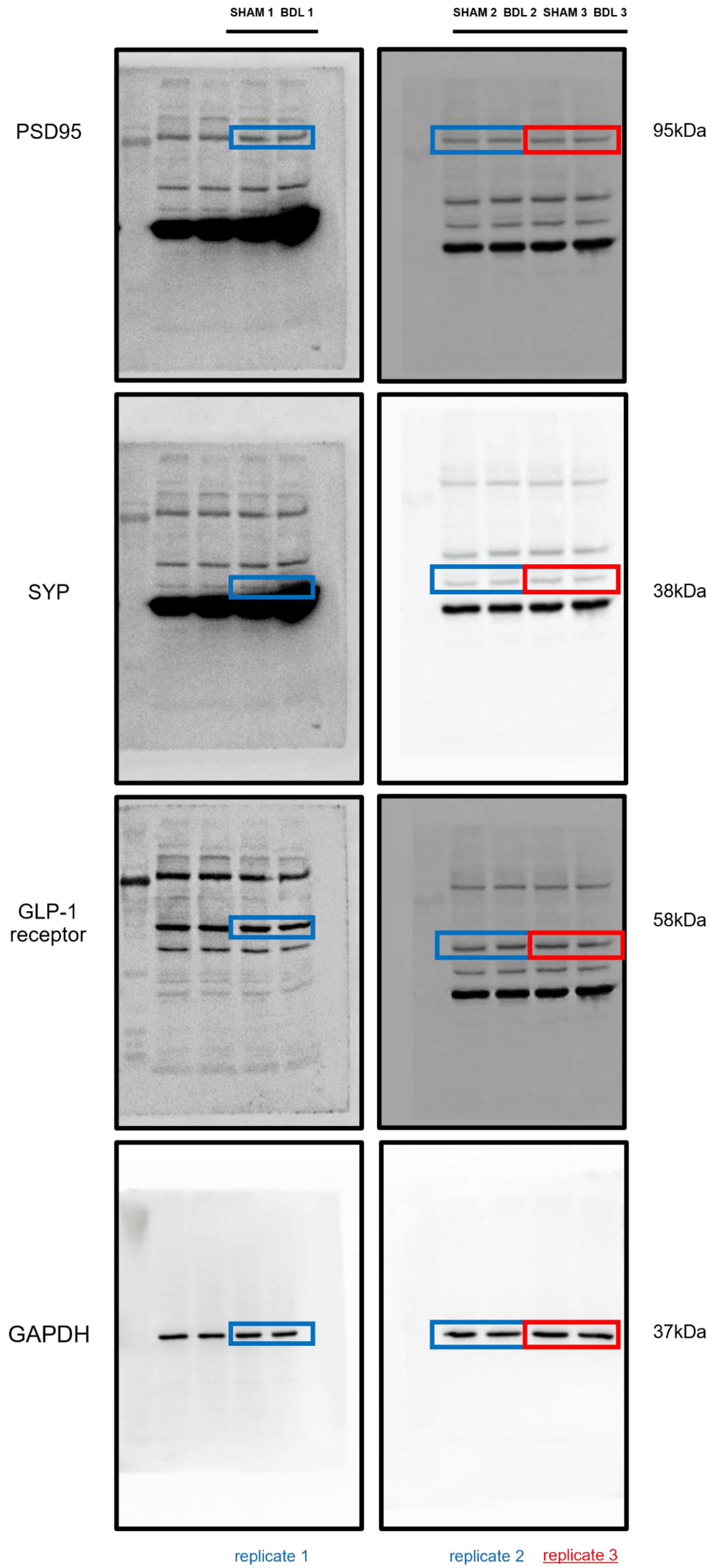

Figure 1G Major

Figure 1G

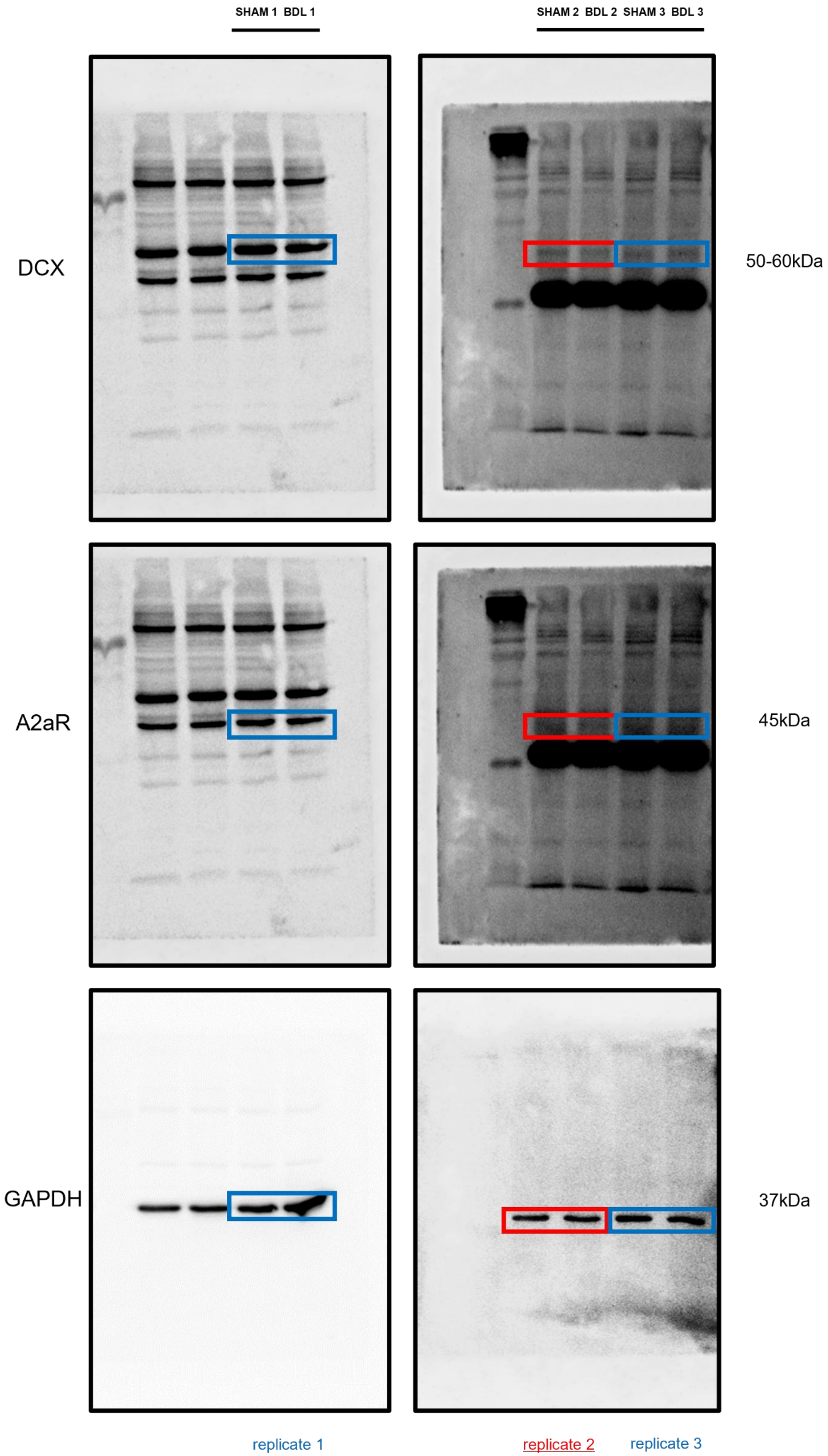

Figure 1G Major

Figure 3E

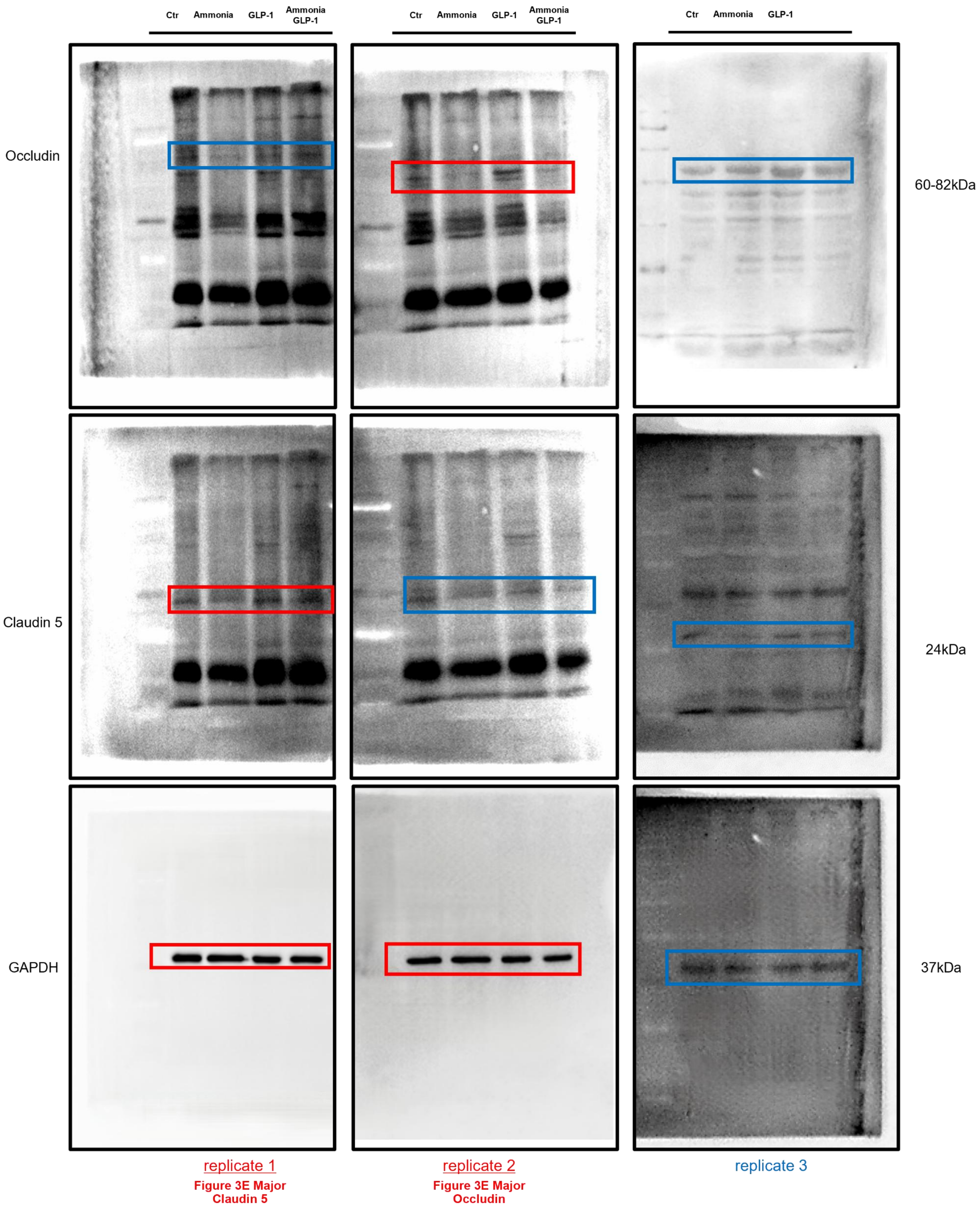
Ctr
Ammonia
GLP-1
Ammonia GLP-1
Ctr
Ammonia
GLP-1
Ammonia GLP-1
Ctr
Ammonia
GLP-1
Occludin
Claudin 5
GAPDH
60-82kDa
24kDa
37kDa
replicate 1
Figure 3E Major Claudin 5
replicate 2
Figure 3E Major Occludin
replicate 3

Figure 3H

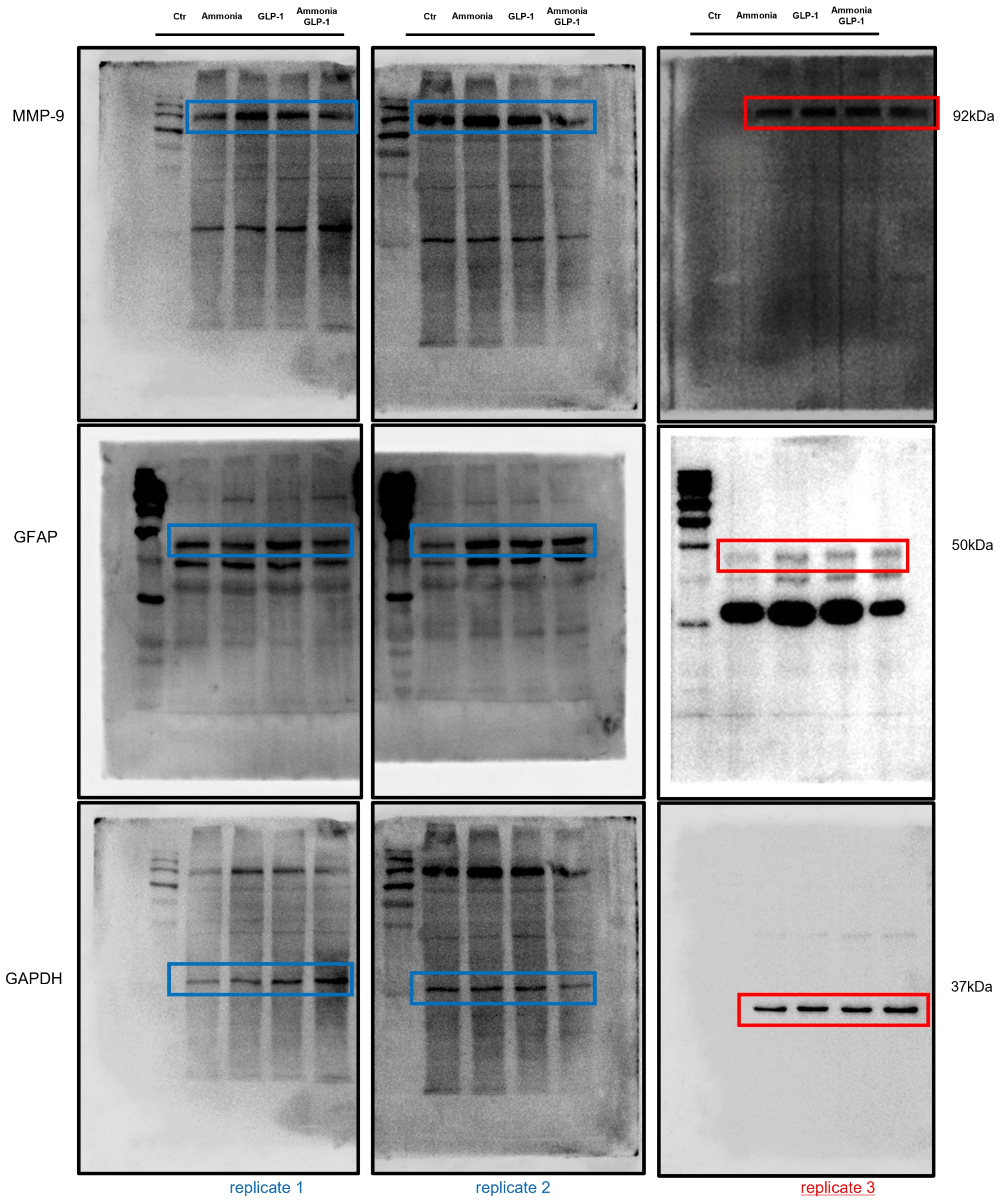

Figure 3H Major

Figure 4E

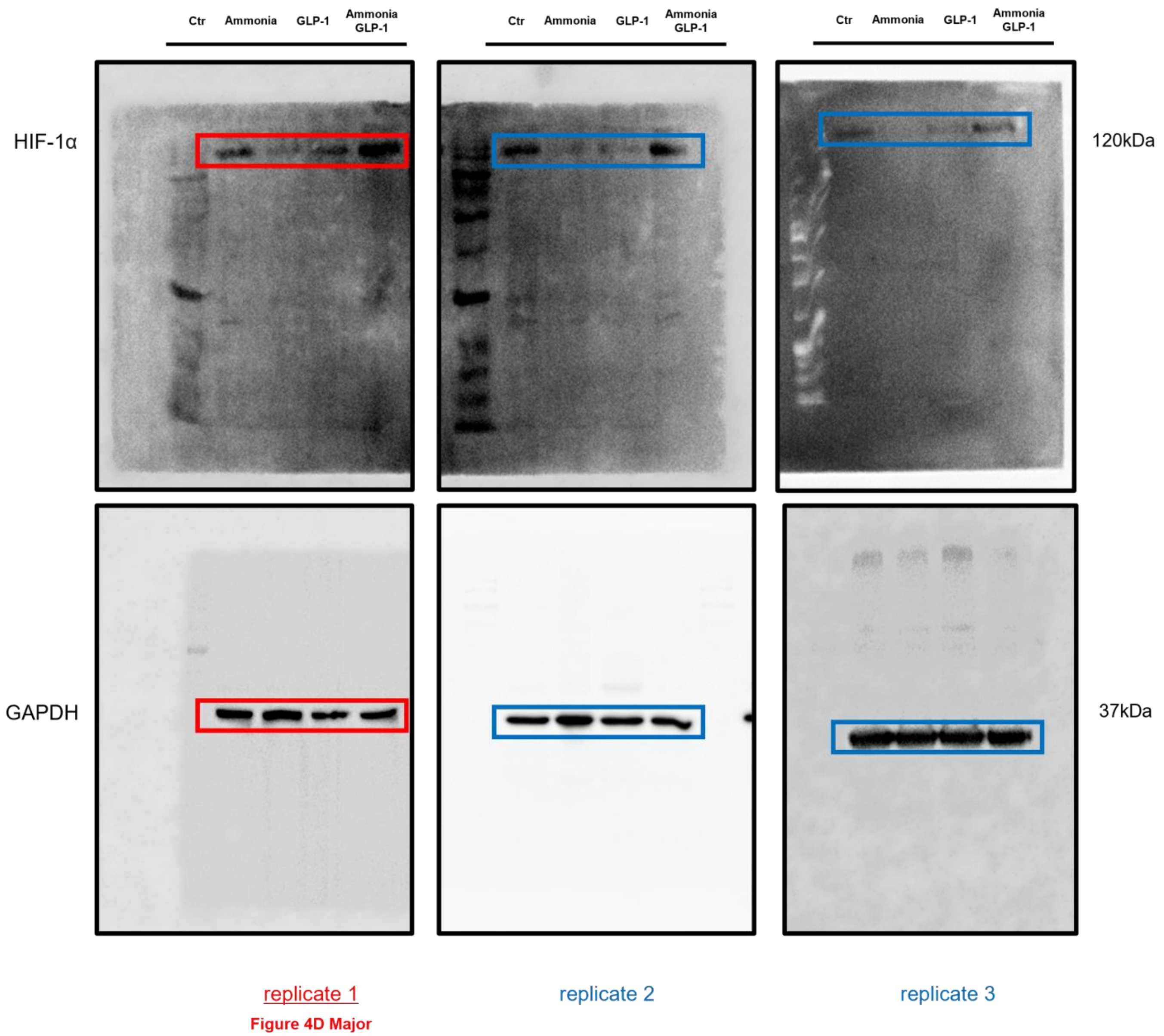

Ctr
Ammonia
GLP-1
Ammonia GLP-1
Ctr
Ammonia
GLP-1
Ammonia GLP-1
Ctr
Ammonia
GLP-1
Ammonia GLP-1
HIF-1α
120kDa
GAPDH
37kDa
replicate 1
Figure 4D Major
replicate 2
replicate 3
