# Supplementary material for: Exendin-4 protects brain endothelial cell damage against hyperammonemic condition
Source: Biochem Biophys Rep. 2026 May 22;46:102644. doi: 10.1016/j.bbrep.2026.102644 (PMC13217415; doi:10.1016/j.bbrep.2026.102644)
Supplement: Multimedia component 3 [file mmc3.docx]

**PCR**

Mouse

| **name** | **Primer sequence** | |
| --- | --- | --- |
| Bcl2 | Fw | GATGACTGAGTACCTGAACCG |
|  | Rev | CAGAGACAGCCAGGAGAAATC |
| Cas9 | Fw | AGCTGGTCACAGACCTTGA |
|  | Rev | CAGCTTCACTACTCTCTGCTC |
| Naip | Fw | CCTGTGGCTGTTGATGCTTTG |
|  | Rev | TCGTCACCTTCTGTGCACTTC |
| Cyp2e1 | Fw | CTTTGCAGGAACAGAGACCA |
|  | Rev | ATGCACTACAGCGTCCATGA |
| Cyp4a1 | Fw | CAACTTGCCCATGATCACACA |
|  | Rev | CATCCTGCAGCTGATCCTTTC |
| mtCo2 | Fw | TCCTCCACTCATGAGCAGTC |
|  | Rev | AACCCTGGTCGGTTTGATGT |
| Tfam | Fw | GTTGTTGGATGGCATGGGTT |
|  | Rev | CGTGTAGAGCTCACGTCTCT |
| Cox7a1 | Fw | GGTCCGGTCTTTTAGCTCAT |
|  | Rev | AGCGTCATGGTCAGTCTGTAC |
| TNF-α | Fw | CTCATGCACCACCATCAAGG |
|  | Rev | ACCTGACCACTCTCCCTTTG |
| IL-1β | Fw | ACTCATTGTGGCTGTGGAGA |
|  | Rev | GCCTGTAGTGCAGTTGTCT |
| IL-6 | Fw | ACTTCACAAGTCCGGAGAGG |
|  | Rev | TGCAAGTGCATCATCGTTGT |
| IL-10 | Fw | AGACCAAGGTGTCTACAAGGC |
|  | Rev | CCAAGGAGTTGTTTCCGTTAGC |
| COX-2  (Ptgs2) | Fw | AAGCCTTCTCCAACCTCTCC |
|  | Rev | GCTGGGCAAAGAATGCAAAC |
| iNOS  (Nos2) | Fw | ATGCGAAAGGTCATGGCTTC |
|  | Rev | CCCAAATGTGCTTGTCACCA |
| eNOS | Fw | GTGACCATAGTGGACCACCA |
|  | Rev | ATGGAAGACAGGAGTTAGGCTG |
| Pex11b | Fw | AACAGATTCGACAACTGGAGG |
|  | Rev | CCATAGGACATTGTCACAGGC |
| H2bc23 | Fw | GTGTACGTGTACAAGGTGCTG |
|  | Rev | GGAGCTGGTGTACTTGGTGA |
| Fkbpl | Fw | AACGAGAAGAACACCGCTCA |
|  | Rev | ACTCCCATCAGGACAGTACCA |
| Pemt | Fw | GTAGCGAGATGGGAGCAGAG |
|  | Rev | TGCATAGAAGCTGGACAGCA |
| Tmem140 | Fw | CAGACAGACGCTGATTTCCT |
|  | Rev | AGACAGAAGTTGTAGAAGCCG |
| Madd | Fw | CTTCAACTCTGCTAACGTGCTG |
|  | Rev | CCGCTGGAAGGCATAGTTAGA |
| Stat2 | Fw | GGAGCAAAGTTCTTGTTCCAC |
|  | Rev | GGTGAACTTGTTCCCAGTCTT |
| Oas3 | Fw | CAGCTGCGATGTGGAACTTG |
|  | Rev | GCGTTGGGCTTCTGTTCAATA |
| GAPDH | Fw | AATGTGTCCGTCGTGGATCT |
|  | Rev | AGACAACCTGGTCCTCAGTG |
